# Hydrophobic Eutectogels Reinforced by Zn^2+^-Coordinated Lignin Nanoparticles for Underwater Wearable Electronics

**DOI:** 10.1007/s40820-026-02302-9

**Published:** 2026-08-03

**Authors:** Fuyuan Lu, Dan Sun, Wei He, Meng Li, Zhengjun Shi, Yiqiang Wu, Shao-Fei Sun, Changyou Shao, Runcang Sun

**Affiliations:** 1 Liaoning Key Laboratory of Lignocellulose Chemistry and Biomaterials, College of Light Industry and Chemical Engineering, Dalian Polytechnic University, Dalian, 116034 People’s Republic of China; 2 College of Chemistry and Materials Engineering, Zhejiang A&F University, Hangzhou, 311300 People’s Republic of China; 3 The Key Laboratory of Forest Resources Conservation and Utilization in the Southwest Mountains of China Ministry of Education, Key Laboratory of National Forestry and Grassland Administration on Biodiversity Conservation in Southwest China, Yunnan Provincial Key Laboratory for Conservation and Utilization of In-Forest Resource, Southwest Forestry University, Kunming, 650224 People’s Republic of China; 4 College of Materials and Chemical Engineering, Southwest Forestry University, Kunming, 650224 People’s Republic of China; 5 College of Materials Science and Engineering, Central South University of Forestry and Technology, Changsha, 410004 People’s Republic of China

**Keywords:** Lignin nanoparticles, Hydrophobic deep eutectic solvents, Underwater adhesion, Underwater communication, Biological motion detection

## Abstract

Wearable flexible sensors for underwater communication and biomotion monitoring are gaining attention. However, developing gel-based strain sensors with high toughness, anti-swelling performance, robust underwater adhesion, and long-term stability remains challenging. Herein, we present a hydrophobic eutectogel (DPF-Zn@LNP-HEG) fabricated via the assembly of a polymerizable hydrophobic deep eutectic solvent (PHDES), 2-phenoxyethyl acrylate (PEA), and Zn^2+^-coordinated lignin nanoparticles (Zn@LNP). The resulting structure, composed of hydrophobic polymer networks and metal-phenolic complexes, forms hydrophobic microdomains that disrupt the hydration layer and prevent water penetration. Meanwhile, Zn@LNP serve as dynamic sacrificial cross-linkers, enhancing the material’s mechanical strength, energy dissipation, and anti-swelling properties. The resulting eutectogel exhibits remarkable tensile strength (1.14 MPa), superior toughness (3.15 MJ m^−3^), excellent anti-swelling properties (< 1% after 30 days), and robust underwater adhesion (1.07 MPa on glass). Based on these properties, we demonstrate an underwater strain sensor with high sensitivity (gauge factor = 8.12) and long-term stability, enabling underwater Morse code transmission, biomotion monitoring, and Bluetooth-based tracking of swimming movements. This work not only provides a versatile design paradigm for multifunctional underwater sensing platforms but also advances the high-value utilization of bio-based materials in next-generation flexible electronics.

**Supplementary Information:**

The online version contains supplementary material available at 10.1007/s40820-026-02302-9.

## Highlights

- A novel deep eutectic solvent with dual functions of hydrophobicity and polymerizability was developed.
- Zn^2+^-coordinated lignin nanoparticles act as dynamic cross-linkers, significantly enhancing the toughness, energy dissipation, and swelling resistance of hydrophobic eutectogels.
- Hydrophobic microdomains formed synergistically by polymer networks and metal-phenolic complexes disrupt the hydration layer on the gel surface, thereby preventing water penetration into the network.

**Supplementary Information:**

The online version contains supplementary material available at 10.1007/s40820-026-02302-9.

## Graphical abstract

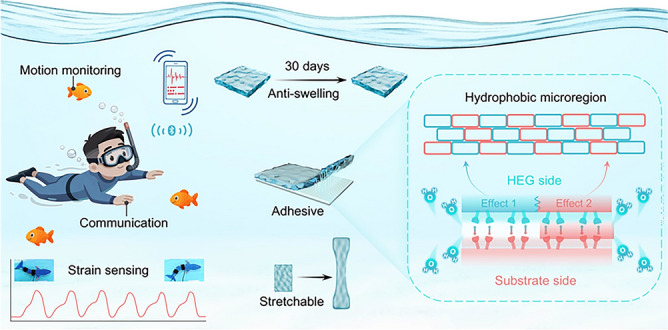

## Introduction

The stability and multifunctional integration of materials in complex aqueous environments remain a significant challenge that restricts technological progress in emerging sectors [1], including flexible electronics [2], underwater operations [3], and marine biological monitoring [4]. Direct aqueous contact or complete immersion can compromise the material’s structural integrity, leading to signal attenuation and unreliable data acquisition [5, 6]. In contrast, gel-based sensors offer a promising alternative, offering advantages such as high biocompatibility, mechanical softness, environmental adaptability, and multifunctional integration over conventional rigid materials [7]. However, the high hydrophilicity of traditional gels significantly limits their use in submerged settings. When immersed, they rapidly absorb water, leading to substantial swelling, mechanical breakdown, and interfacial failure, which negatively impacts their functionality in waterproof electronics and marine sensors [8, 9]. In contrast, hydrophobic gels, known for their low surface energy and exceptional swelling resistance, present a strong solution to overcome these challenges. These materials provide a foundation for the development of reliable and durable sensing platforms capable of operating effectively in demanding aquatic environments [10].

Since their introduction in 2015 [11], hydrophobic deep eutectic solvents (HDES) have developed rapidly, leading to significant advancements in a wide range of applications, including microextraction [12, 13], heavy metal removal [14], CO_2_ capture [15], and flexible electronics [16]. The initial HDES formulations typically consisted of decanoic acid as the hydrogen bond donor (HBD) and long-chain quaternary ammonium salts, such as tetraoctylammonium bromide, methyltrioctylammonium bromide, or tetraheptylammonium chloride, serving as the hydrogen bond acceptor (HBA). Another class of HDES is formed by combining two neutral hydrophobic compounds, typically involving a natural hydrophobic HBA (e.g., menthol, carvacrol) and specific hydrophobic HBD (e.g., long-chain carboxylic acids or alcohols). The overall hydrophobicity of an HDES is determined by the intrinsic hydrophobicity of its components. Typically, both the HBA and HBD need to be hydrophobic, or one strongly hydrophobic component may dominate the system’s behavior [17]. Recently, hydrophobic eutectogels (HEG), created from HDES and hydrophobic polymers, have emerged as promising materials for applications such as drug delivery [18] and flexible sensing [16]. However, in many reported cases, the HDES acts only as a solvent, leading to compromised network stability and suboptimal mechanical properties [16, 19]. Polymerizable HDES (PHDES) addresses this issue by incorporating reactive groups, such as vinyl functionalities, that enable covalent polymerization and precise structural control [20]. This strategy allows for the rational molecular design of PHDES, enabling fine-tuning of key factors such as hydrophobicity and cross-linking density, offering a versatile approach for customizing material performance [21]. HEG derived from PHDES exhibit improved hydrophobicity, enhanced mechanical strength, and superior functional integration, making them promising for use in demanding aqueous environments. However, research on these systems remains in its infancy, and their full potential for structural reinforcement and multifunctional applications remains largely untapped.

Lignin, the most abundant aromatic biomass in nature, offers significant advantages such as wide availability, low cost, and excellent biocompatibility [22]. Lignin nanoparticles (LNP), derived from this renewable source, exhibit remarkable reactivity and a high specific surface area, making them promising sustainable functional fillers for polymer systems [23–25]. For example, Yang et al. showed that incorporating the optimal amount of LNP enhanced the thermal and mechanical properties of polyvinyl alcohol (PVA)/chitosan (Ch) gels. This improvement was attributed to strong interfacial interactions between the nanoparticles and the polymer matrix [26]. Additionally, when LNP were chelated with metal ions (e.g., Ag^+^, Ca^2+^, Fe^3+^), dynamic metal coordination bonds formed, acting as effective cross-links within the gel network, thus boosting mechanical strength and structural stability [27]. Cui et al. demonstrated that modifying LNP with silver nanoparticles (Ag NPs) created a synergistic reinforcement effect, leading to robust, fatigue-resistant gels, and offering a new approach for utilizing LNP as high-performance functional fillers [28]. Wang et al. reported a catalytic system using LNP-Fe^3+^-ammonium persulfate (APS) to fabricate poly(2-hydroxyethyl methacrylate)/LNP/Ca^2+^ (PHEMA/LNP/Ca) gels. These composites exhibited exceptional stretchability, strong adhesion, and efficient self-healing, which were enabled by a combination of metal coordination, hydrogen bonding, and other synergistic interactions [29]. Furthermore, the aromatic structure of lignin facilitated π-π stacking with hydrophobic polymer segments, reinforcing the network and improving overall hydrophobicity. This combination presents an effective strategy for designing anti-swelling gels that maintain stable performance in aqueous environments [30].

**Fig. 1:**
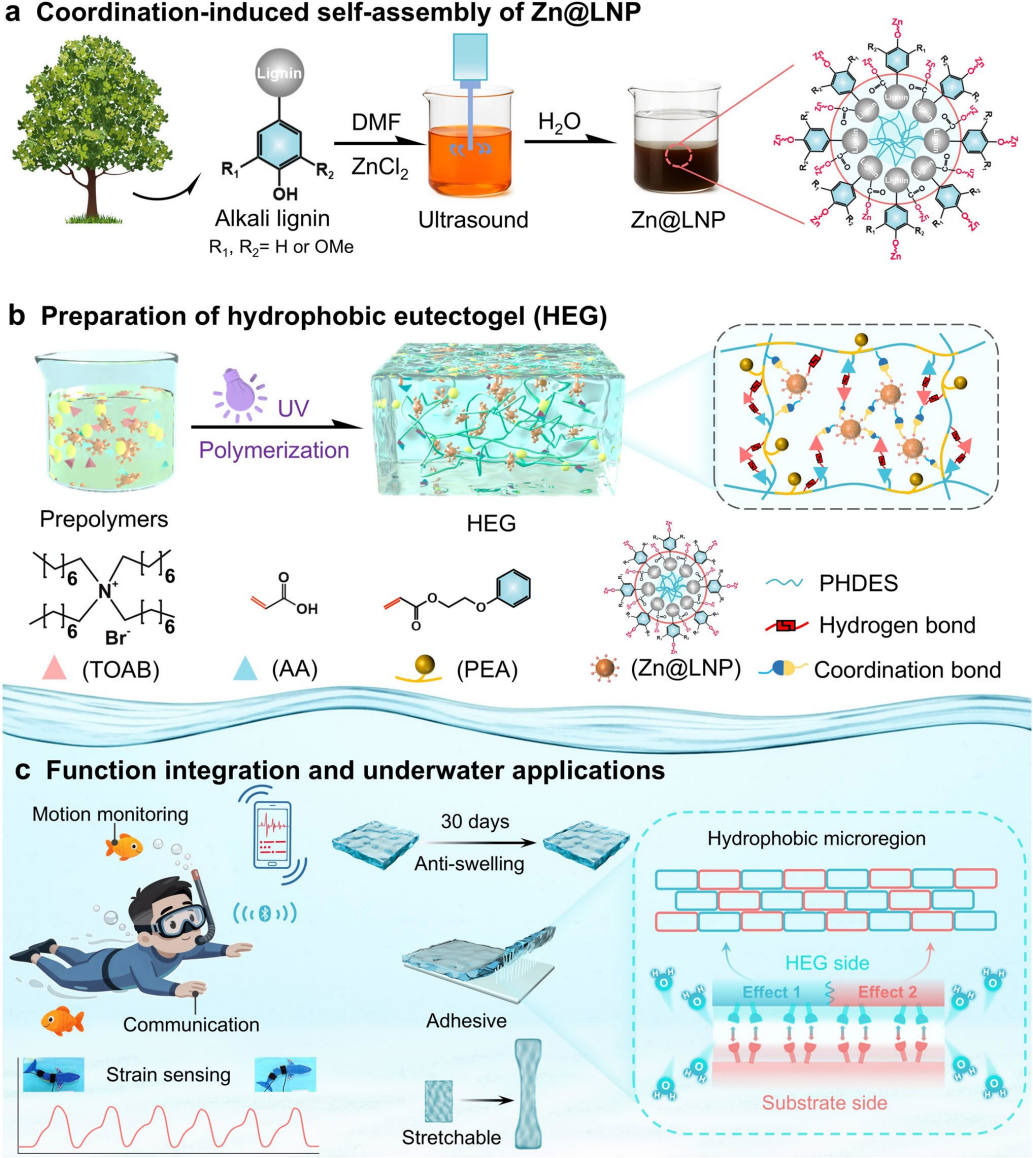
**a** Schematic diagram of Zn@LNP fabrication. **b** Schematic diagram of HEG preparation via one-pot synthesis. **c** Functional integration and underwater applications of HEG

Currently, the development of hydrophobic gels faces three major challenges: first, constructing a composite network that combines strong hydrophobicity with molecular-level compatibility to prevent phase separation and performance deterioration; second, balancing mechanical strength and anti-swelling performance to achieve long-term stability in sustained underwater environments; and third, integrating mechanical strength, underwater adhesion, and responsive sensing to meet the stringent demands of next-generation flexible electronics for multifunctional integration. To address these challenges, we designed and synthesized a polymerizable hydrophobic deep eutectic solvent (PHDES) using acrylic acid (AA) as the hydrogen bond donor (HBD) and tetraoctylammonium bromide (TOAB) as the hydrogen bond acceptor (HBA). Through molecular design, this PHDES is endowed with both inherent hydrophobicity and polymerizability and exhibits excellent compatibility with the hydrophobic monomer 2-phenoxyethyl acrylate (PEA). Subsequently, we employed a synergistic approach combining ultrasonication and anti-solvent techniques to synthesize Zn^2+^-coordinated lignin nanoparticles (Zn@LNP) (Fig. 1a). The hydroxyl, carboxyl, and Zn^2+^-coordinating groups on the nanoparticle surface form reversible hydrogen bonds and coordination interactions with polymer chains, enhancing the gel network and improving energy dissipation. Additionally, *N,N*-dimethylformamide (DMF) was incorporated to enhance interfacial adhesion. Through a one-pot UV-initiated polymerization process, we successfully synthesized a hydrophobic eutectogel (HEG; Fig. 1b). This unique structural design enables the formation of hydrophobic microdomains via the synergistic interaction between metal-polyphenol complexes and hydrophobic polymer networks. These microdomains disrupt the hydration layer and prevent water penetration. Meanwhile, Zn@LNP act as cross-linkers, significantly enhancing the material’s mechanical strength, energy dissipation, and anti-swelling properties. Furthermore, DMF endows HEG with excellent chain segment flexibility, enabling better conformity to the microscale morphology of substrate surfaces, thereby increasing effective interfacial contact area and interaction sites. Through these synergistic effects, HEG achieves a combination of high mechanical strength, excellent anti-swelling performance, robust underwater adhesion, and a sensitive electromechanical response. These integrated properties position HEG as a promising platform for smart underwater wearable sensors, with potential applications in underwater communication, biological motion monitoring, and Bluetooth-enabled swimming activity tracking (Fig. 1c).

## Experimental Section

### Materials

Alkali lignin (AL) was provided by Shandong Longlive Biotechnology Co., Ltd (Shandong, China). The AL was purified through acid treatment, followed by centrifugation, repeated washing with deionized water, and freeze-drying. Tetraoctylammonium bromide (TOAB, 98%), acrylic acid (AA, 99%), 2-phenoxyethyl acrylate (PEA, 90%), 2-hydroxy-4’-(2-hydroxyethoxy)-2-methylpropiophenone (I2959, 98%), *N,N*-dimethylformamide (DMF, 99.5%), zinc chloride (ZnCl_2_, 98%), aluminum chloride (AlCl_3_), magnesium chloride (MgCl_2_), calcium chloride (CaCl_2_), sodium hydroxide (NaOH, 98%), and sodium chloride (NaCl) were purchased from Shanghai Macklin Co., Ltd (Shanghai, China). Iron(III) chloride (FeCl_3_) was obtained from Shanghai Aladdin Biochemical Technology Co., Ltd (Shanghai, China). Hydrochloric acid was obtained from Guangzhou Chemical Reagent Factory, China. All chemical reagents were received without any further purification. Deionized water (DI) used in this work was produced using a laboratory water purification system. Natural seawater was collected from the coastal area of Dalian, China.

### Preparation of PHDES

The polymerizable hydrophobic deep eutectic solvent (PHDES) was prepared by mixing AA and TOAB at a molar ratio of 3:1. The mixture was stirred in a sealed vessel at room temperature for 1 h until a transparent, light-yellow liquid formed (Fig. S1).

### Preparation of Zn@LNP

Zn^2+^-coordinated lignin nanoparticles (Zn@LNP) were synthesized using an ultrasound-assisted anti-solvent method. Briefly, ZnCl_2_ and AL at a mass ratio of 1:1 were first dissolved in DMF to prepare a homogeneous mixed solution with a concentration of 10 mg mL^−1^, followed by ultrasonic treatment in an ice-water bath for 1 h to avoid overheating. Thereafter, DI was added dropwise into the mixture as the nanoparticle precipitation occurred. The precipitates were collected by centrifugation and thoroughly washed with DI to remove residual ZnCl_2_ and DMF. The final products were obtained by freeze-drying for subsequent experiments and characterization. By adjusting the concentration of the mixed solution (5, 10, and 20 mg mL^−1^) while keeping all other preparation parameters identical, Zn@LNP with different particle sizes were prepared. Furthermore, Fe@LNP, Al@LNP, Mg@LNP, and Ca@LNP were prepared using the same method for comparison.

### Preparation of DPF-Zn@LNP-HEG

The D_2_P_3_F_4_-Zn@LNP-HEG was synthesized as a representative example utilizing a facile one-pot strategy. Initially, 10 mg Zn@LNP (0.2 wt% for the total monomers) was dispersed in a mixture of 2.0 g PHDES, 3.0 g PEA, 0.2 g DMF, and 52 mg I2959 and then stirred for 1h until a homogeneous solution formed. Subsequently, the mixture was photopolymerized into a eutectogel under UV light. For comparison, other eutectogels were prepared using the identical procedure as described above. The resulting eutectogels are labeled as DxPyFz-Zn@LNPn-HEG, where x and y denote the mass ratios of PHDES to PEA (3:1, 3:2, 1:1, 2:3, and 1:3); z indicates the DMF content as a weight fraction of the total monomers (2, 4, and 6 wt%); n indicates the Zn@LNP content as a weight fraction of the total monomers (0.1, 0.2, 0.3, and 0.4 wt%). The photoinitiator I2959 was added at 1 wt% relative to the total monomer mass. To simplify the nomenclature, all subsequent HEG samples containing 0.2 wt% Zn@LNP are referred to as “DPF-Zn@LNP-HEG” (where D, P, and F represent PHDES, PEA, and DMF, respectively).

## Results and Discussion

### Preparation and Characterization of the PHDES

**Fig. 2:**
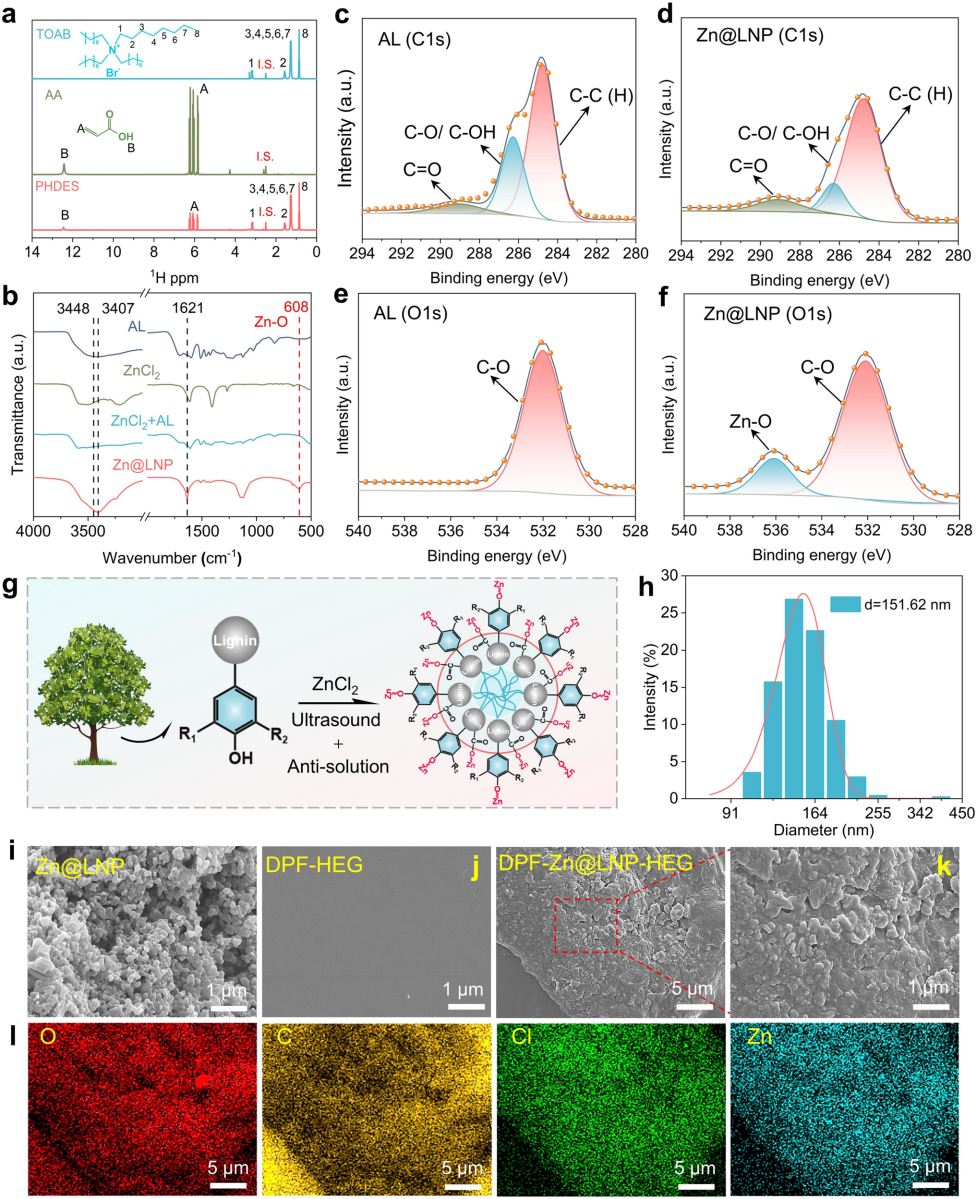
**a** ^1^H NMR spectra of TOAB, AA, and PHDES. **b** FTIR spectra of AL, ZnCl_2_, ZnCl_2_ + AL, and Zn@LNP. High-resolution XPS spectra of C 1*s* **c, d** and O 1*s* **e, f** regions for AL **c, e** and Zn@LNP **d, f. g** Schematic illustration of the Zn@LNP self-assembly process. **h** Particle size distributions of Zn@LNP. Cross-sectional SEM images of **i** Zn@LNP, **j** DPF-HEG, and **k** DPF-Zn@LNP-HEG, with **l** EDS elemental mappings of O, C, Cl, and Zn

In this study, the HEG was constructed through the formation of an interpenetrating network between PHDES and the hydrophobic polymer. To fabricate this unique gel material, a novel PHDES was first designed and synthesized using AA as the HBD and TOAB as the HBA. The two components were mixed and stirred at a molar ratio of 3:1 under ambient conditions, yielding a light-yellow transparent liquid (Fig. S1). In this system, AA provides polymerizable vinyl groups, while TOAB imparts hydrophobicity (Fig. S2). The successful formation of PHDES was confirmed by ^1^H nuclear magnetic resonance (^1^H NMR) and differential scanning calorimetry (DSC) analyses. The ^1^H NMR spectrum showed a downfield shift of the carboxylic proton signal of AA from *δ* 12.42 ppm in the pure monomeric state to *δ* 12.45 ppm in the PHDES (Figs. 2a and S3a), indicating a deshielding effect induced by hydrogen bonding between AA and the bromide ions (Br^–^) of TOAB. The positions of other characteristic peaks remained unchanged, and no extraneous signals were detected, confirming that no side reactions occurred during the preparation. This result demonstrates that the interaction between AA and TOAB is exclusively mediated by hydrogen bonding. DSC analysis further supported the formation of the eutectic mixture (PHDES). As illustrated in Fig. S3b, pure AA and TOAB exhibited sharp melting peaks at 14.7–50.8 °C, respectively, whereas the PHDES showed a single melting peak near 0 °C, which is markedly lower than those of its individual components, a characteristic feature of deep eutectic behavior [31]. This confirms the successful synthesis of the PHDES. Notably, the vinyl group on the AA chain endows the PHDES with polymerizable capability, laying the foundation for the subsequent interpenetrating network. The hydrophobic nature of the PHDES was visually confirmed by its phase separation behavior upon contact with water (Movie S1).

### Preparation and Characterization of the Zn@LNP

Zn@LNP was prepared using a strategy that combines ultrasound and anti-solvent methods. The synthesis process involved two steps: First, ultrasound treatment was employed to promote coordination between Zn^2+^ and alkali lignin (AL); then, an anti-solvent method was used to induce Zn@LNP self-assembly (Fig. 1a). To elucidate the interactions between AL and ZnCl_2_, Fourier transform infrared spectroscopy (FTIR) and X-ray photoelectron spectroscopy (XPS) were performed. FTIR analysis provided clear evidence of coordination (Fig. 2b). In the spectrum of AL, the characteristic hydroxyl groups (–OH) stretching vibration peak was observed at 3448 cm^–1^. This peak underwent a noticeable redshift to 3407 cm^–1^ in Zn@LNP, suggesting the involvement of hydroxyl oxygen atoms in coordination with Zn^2+^. Additionally, the spectrum of Zn@LNP displayed an asymmetric carboxylate stretching vibration at 1621 cm^–1^ (v_as_ (COO^–^)) and a Zn–O stretching vibration at 608 cm^–1^ (v_as_ (Zn–O)). These observations confirm the formation of coordination bonds between Zn^2+^ and both –COOH and –OH in AL [32, 33]. XPS analysis further supported these findings. The C 1*s* spectrum of pure AL displayed three distinct peaks corresponding to C–C/C–H (284.8 eV), C–O/C–OH (286.0 eV), and C=O (289.0 eV) bonds, while the O 1*s* spectrum featured a single dominant peak at 532.0 eV, assigned to C–O bonding (Fig. 2c, d). In contrast, the C 1*s* spectrum of Zn@LNP exhibited slight shifts in the binding energies of the C–O/C–OH and C=O peaks. Moreover, a new peak emerged at approximately 536.1 eV in the O 1*s* spectrum, attributed to Zn–O bonding (Fig. 2e, f), reinforcing the occurrence of coordination between Zn^2+^ and oxygen-containing functional groups in AL [34]. These findings align well with the FTIR results, collectively confirming the coordination interaction between Zn^2+^ and AL in Zn@LNP. Control experiments (Fig. S4) showed that the mixture of ZnCl_2_ and AL did not exhibit the characteristic peak shifts or newly emerged signals observed in Zn@LNP, ruling out mere physical adsorption or encapsulation between Zn^2+^ and AL in Zn@LNP. These results provide critical structural evidence supporting the successful fabrication of the material and lay the foundation for subsequent functional studies. Meanwhile, comparison of the FTIR spectra of Fe@LNP, Al@LNP, Mg@LNP, and Ca@LNP revealed that the coordination interactions between Mg^2+^/Ca^2+^ and AL are relatively weak and exist mainly via physical blending, which is primarily attributed to their strong hydration effects hindering contact with the oxygen-containing groups of AL. Al^3+^ exhibited partial coordination with the –OH of AL. Furthermore, for Fe@LNP, the C=O characteristic peak at 1705 cm^–1^ was significantly weakened, while new absorption peaks appeared at 1654 cm^–1^ (aromatic C=C) and 662 cm^–1^ (Fe–O) [35], indicating the formation of coordination between Fe^3+^ and AL (Fig. S5). These results provide a theoretical foundation for further investigation of metal-coordinated LNP with different metal ions and their reinforcement mechanisms in HEG.

Dynamic light scattering (DLS) and scanning electron microscopy (SEM) analyses revealed that Zn@LNP exhibit a uniform quasi-spherical morphology with an average diameter of approximately 151.62 nm (Fig. 2h, i), which is about one order of magnitude smaller than the average diameter of raw AL (1218.6 nm, Fig. S6), confirming the successful nanosizing of AL. The self-assembly process of Zn@LNP is illustrated in Fig. 2g. Comparative analysis of the eutectogel cross-sectional morphology before (Fig. 2j) and after Zn@LNP incorporation (Fig. 2k) revealed significant structural transformation: the originally smooth and compact gel matrix became populated with uniformly distributed nanoparticles with sizes ranging from 100 to 200 nm. Energy-dispersive spectroscopy (EDS) elemental mappings of DPF-Zn@LNP-HEG (Figs. 2l and S7, Table S1) showed that C, O, Cl, and Zn are evenly distributed throughout the eutectogel matrix.

### Preparation and Characterization of the HEG

The high-performance HEG was synthesized by intertwining PHDES with PEA, forming a dense hydrophobic network. The molecular architecture of PEA, comprising polar ester groups (COO^–^) and hydrophobic phenoxy chains (O–C_6_H_5_), enables it to form diverse physical cross-linking interactions with PHDES. Specifically, as shown in Fig. S8, in the interaction system between PHDES and PEA, the introduction of PEA led to the appearance of new characteristic absorption peaks at 1600, 1450, and 1230 cm^–1^, corresponding to the skeletal vibrations of the benzene ring and the characteristic signals of aromatic ether bonds. Meanwhile, the C=O stretching vibration peak at 1730 cm^–1^ and the quaternary ammonium salt C–H characteristic peaks at 2850–2920 cm^–1^ exhibited blueshifts. These phenomena indicated that PEA generated a significant hydrophobic effect, disrupting the original hydrogen bonding network in the system and weakening the strong intermolecular interactions among polymer chains, confirming that its flexible phenoxyethyl side chains act as soft segments, imparting flexibility to the HEG system. At the same time, these synergistic interactions enable the formation of a tightly woven hydrophobic layer between PEA and PHDES, effectively hindering water molecule infiltration and improving the overall water resistance of the HEG system [36]. Experimental data in Fig. S9 clearly demonstrated that the surface contact angle of HEG increased significantly with increasing PEA content, confirming the enhancement in hydrophobicity of the network. Furthermore, a new C=O characteristic peak at 1670 cm^–1^ was attributed to the presence of a trace amount of DMF in the system, indicating that DMF mainly exists as a solvent within the HEG system. After the introduction of Zn@LNP, the asymmetric stretching vibration peak of the carboxylate group (v_as_ (COO^–^)) shifted from 1621 cm^–1^ in pristine Zn@LNP to 1648 cm^–1^ in the DPF-Zn@LNP-HEG system. This result confirms the formation of metal coordination interactions between Zn@LNP and the carboxyl groups in the eutectogel network, and such coordination anchors Zn@LNP as physical cross-linking points within the gel network, further constructing a stable cross-linked structure. A one-pot UV-initiated bulk polymerization method was employed, in which Zn@LNP were copolymerized with PHDES and PEA in DMF. This process not only constructed a covalently cross-linked network but also introduced non-covalent interactions via intermolecular hydrogen bonding and coordination bonds, thereby yielding a polymer matrix with high mechanical strength, flexibility, and strong adhesion.

Notably, PHDES and PEA demonstrate exceptional mutual miscibility, a critical prerequisite for the fabrication of high-performance HEG. In addition, the low eutectic character of PHDES allows it to remain liquid at ambient temperature, providing an ideal medium for PEA permeation and diffusion. This ensures molecular-level mixing of the two components, which is essential for forming a uniform HEG network. Regarding optical properties, the synthesized DPF-HEG displayed high transparency, achieving approximately 95% transmittance across the visible spectrum and appearing nearly colorless. Even after incorporating 0.2 wt% Zn@LNP, the resulting DPF-Zn@LNP-HEG still retained high transparency (~ 83%), allowing clear visualization of underlying images (Fig. S10). This optical clarity is particularly advantageous for applications that require both functional performance and visual access.

### Mechanical Properties

In applications spanning flexible electronics, biomedical engineering, and extreme-environment devices, the mechanical properties of gel materials are crucial to their practical application [37–39]. Owing to its hydrophobic adaptability and unique network structure, HEG offers notable advantages in applications such as marine detection sensors and waterproof flexible devices [19, 40]. To ensure stable sensor output, the mechanical properties of HEG were systematically investigated. The study began by evaluating the mechanical behavior of DP-HEG samples with varying PHDES-to-PEA mass ratios. As illustrated in Fig. 4a, the incorporation of PEA, which contains flexible phenoxyethyl side chains, into the rigid PHDES network introduces molecular-level flexibility as soft segments. This molecular-level flexibility reduces intermolecular interactions between polymer chains, thereby increasing the fracture extensibility of DP-HEG and enabling easier deformation. The elongation at break of DP-HEG increased with increasing PEA content. However, when the PEA content exceeded an optimal threshold, it caused fragmentation of the cross-linked network and excessive plasticization, leading to a ductile-to-brittle transition. DP-HEG achieved a maximum elongation at break of 419% at a PHDES/PEA mass ratio of 2:3.

As a polar aprotic solvent, DMF features polar functional groups (e.g., carbonyl group, C=O) that interact with the polar segments of the HEG network, thereby enhancing segmental flexibility (Fig. S11). Such improved deformability enables HEG to better conform to the microscale morphology of substrate surfaces, increasing interfacial contact and intermolecular interactions. This consequently strengthens the adhesion performance and expands the application potential of HEG. However, excessive DMF content weakens intermolecular interactions and reduces the cross-linking density between monomers, resulting in over-solvation of polymer chains. This phenomenon impairs the ability of HEG to dissipate energy via cooperative deformation, ultimately reducing the elongation at break [41]. Optimization results indicated that incorporating 4 wt% DMF increased the elongation at break of HEG to 557.8%, representing a 32.9% enhancement compared to DP-HEG (Fig. 4b). To further enhance HEG mechanical properties, Zn@LNP was incorporated as reinforcing and cross-linking agents, leading to notable performance improvements. The effect of different Zn@LNP loadings on the mechanical properties of HEG was systematically investigated (Fig. 4c, d). At the optimal Zn@LNP concentration of 0.2 wt%, HEG exhibited maximum tensile stress (1.14 MPa), elongation at break (667.6%), Young’s modulus (0.71 MPa), and toughness (3.15 MJ m^–3^). To further clarify the role of nanoscale fillers in the mechanical properties of HEG, we conducted a series of control experiments. As compared with raw AL, LNP possesses a high specific surface area and highly accessible surface functional groups, which enables the formation of abundant non-covalent cross-linking points between per unit mass of filler and the polymer matrix, thereby significantly improving the mechanical strength and toughness of HEG (Fig. S12). Meanwhile, we experimentally elucidated the individual and synergistic effects of Zn^2+^ and LNP (Fig. S13). The addition of ZnCl_2_ alone increases the cross-linking density of HEG via coordination interactions, thereby enhancing both its strength and elongation at break. In contrast, the introduction of LNP alone mainly improves the mechanical strength of HEG through physical filling and interfacial interactions. When Zn^2+^ coordinates with LNP, a clear synergistic effect occurs, strengthening the interfacial interactions between Zn@LNP and the HEG matrix and ultimately improving both the strength and toughness of HEG. Furthermore, as the particle size of Zn@LNP increased (Fig. S14a, b), the toughness of HEG first increased and then decreased (Fig. S15a, b). The reason is that the excessively large particle sizes lead to poor dispersion in the matrix, and the local volume effect disrupts the uniformity of the network structure. This enhancement is mainly attributed to the following aspects: First, Zn@LNP exhibits excellent dispersion stability in the HEG liquid precursor (Fig. S16). Second, hydrogen bonds formed between the hydroxyl and carboxyl groups on the Zn@LNP surface and the HEG network introduce additional physical cross-linking points, thereby increasing the effective cross-link density and promoting uniform stress distribution through the nanofiller effect. Meanwhile, the coordination bonds between Zn^2+^ in Zn@LNP and the oxygen-containing groups in HEG serve as sacrificial bonds, providing reversible extensibility under mechanical stress and allowing polymer chain slippage [28]. To verify whether other metal ions can achieve similar effects, 0.2 wt% of Fe@LNP, Al@LNP, Mg@LNP, and Ca@LNP were added, respectively, to the HEG. As shown in Fig. S17, the introduction of Fe@LNP and Al@LNP formed poorly reversible chemical bonds with the HEG through strong coordination interactions, which hindered energy dissipation and consequently reduced the flexibility of the HEG. In contrast, due to the weak coordination strength of Mg@LNP and Ca@LNP, the mechanical properties of the resulting HEG were all inferior to those of DPF-Zn@LNP-HEG. Thus, it can be seen that Zn^2+^ benefits from its moderate Lewis acidity and flexible coordination geometry, enabling the formation of dynamic reversible coordination bonds with oxygen-containing ligands. Although Fe^3+^, Al^3+^, Mg^2+^, and Ca^2+^ also exhibit similar reinforcement mechanisms, their overall effects are inferior to those of Zn^2+^.

**Fig. 3:**
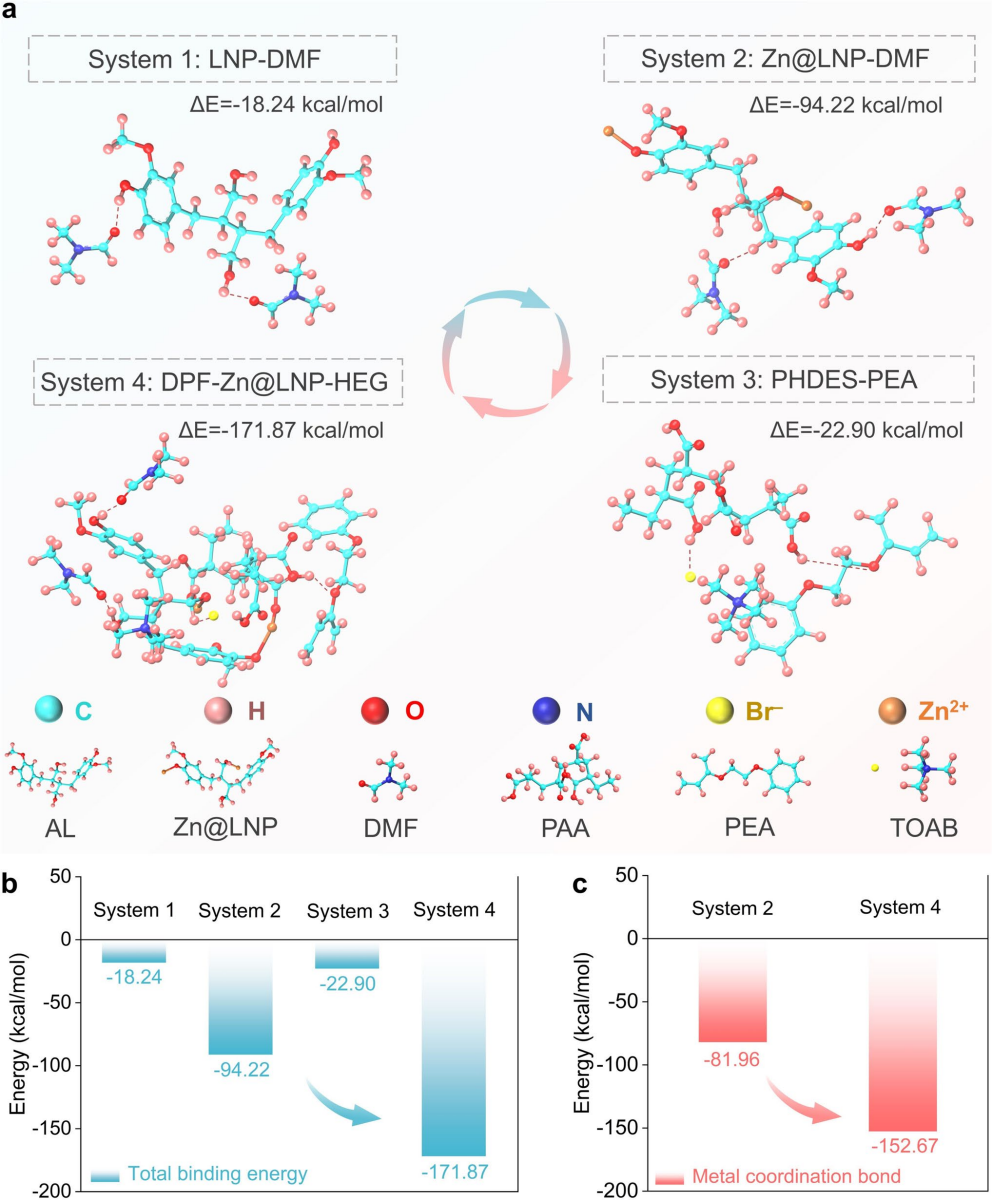
**a** Optimized structural models of LNP-DMF, Zn@LNP-DMF, PHDES-PEA, and DPF-Zn@LNP-HEG. **b** Calculated total binding energies and **c** metal coordination bonds of the above molecular systems

**Fig. 4:**
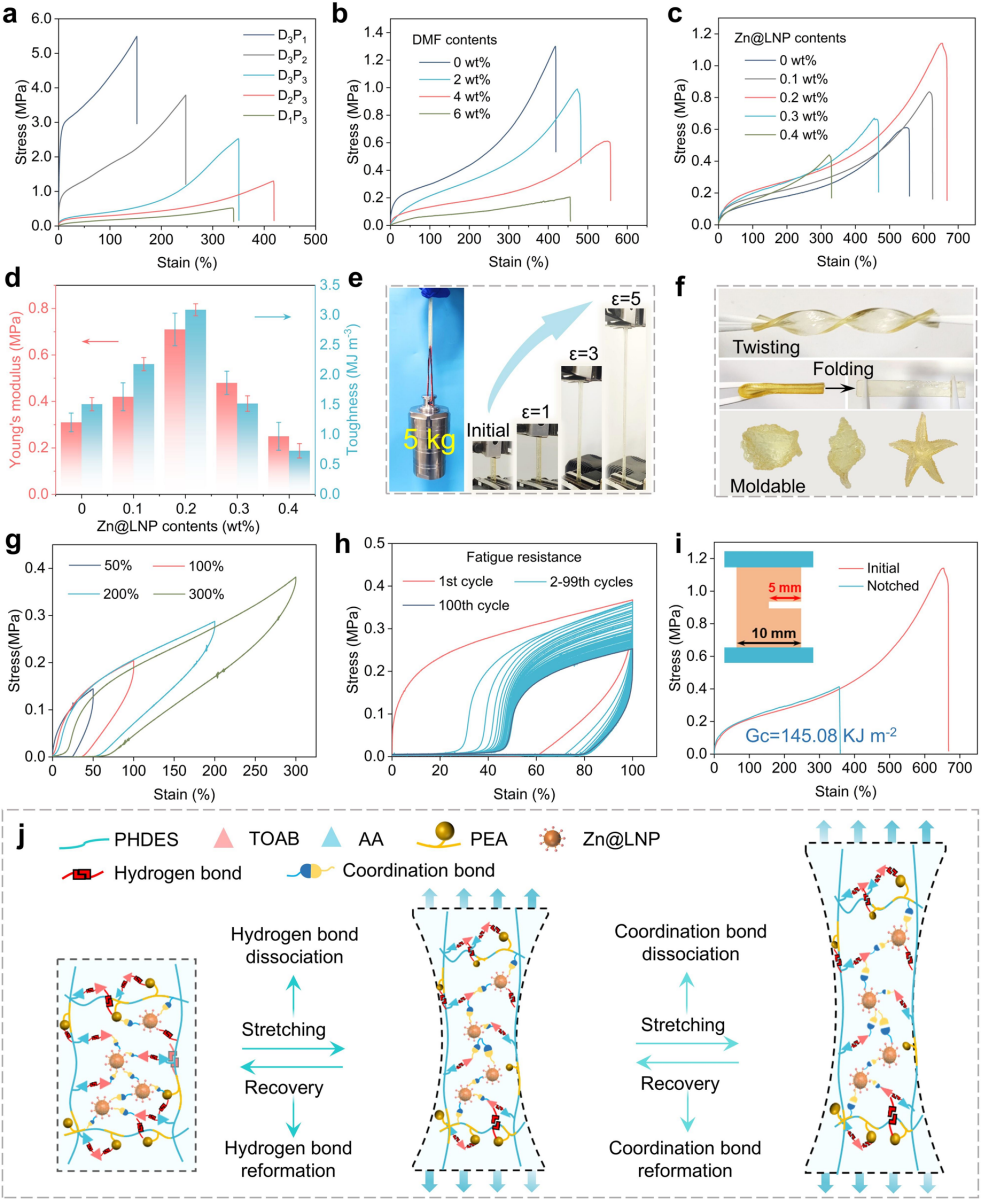
Mechanical properties of DPF-Zn@LNP-HEG. **a–c** Stress–strain curves with varying PHDES/PEA mass ratios (for DP-HEG), DMF contents (for DPF-HEG), and Zn@LNP contents. **d** Young’s modulus and toughness as a function of Zn@LNP contents. **e** Photographs demonstrating high mechanical strength. **f** Photographs showing moldability, torsion behavior, and foldability with full recovery. **g** Tensile loading–unloading curves at different strains. **h** Cyclic tensile loading–unloading curves over 100 cycles at 100% strain. **i** Stress–strain curves of unnotched and notched samples. **j** Schematic of the structural evolution in the DPF-Zn@LNP-HEG network

Subsequently, density functional theory (DFT) calculations were employed to elucidate the intermolecular interaction mechanisms in the HEG system, with a focus on the binding energies of LNP-DMF (system 1), Zn@LNP-DMF (system 2), PHDES-PEA (system 3), and DPF-Zn@LNP-HEG (system 4) (Fig. 3a). The local binding trends revealed by these computational results indicate that metal coordination plays a key role in stabilizing the LNP-solvent interface, facilitating a more energetically favorable configuration and stabilizing the composite system (Fig. 3b, c). This further highlights the core mechanism by which Zn@LNP reinforces the HEG by stabilizing the network structure, enhancing mechanical strength, and improving fatigue resistance. When Zn@LNP content was excessive, its abundant surface phenolic –OH and aromatic rings, known for strong radical-scavenging activity, may excessively quench free radicals, thereby inhibiting the formation of covalent cross-links in the gel network and leading to a loosely structured three-dimensional matrix [42]. Based on these findings, DPF-Zn@LNP-HEG fabricated at a PHDES: PEA mass ratio of 2:3 with 4 wt% DMF and 0.2 wt% Zn@LNP was selected for subsequent studies. As illustrated in Fig. 4e, the HEG displayed excellent tensile properties, withstanding strains of up to 600% without fracture. It also supported a 5.0 kg weight without failure, clearly demonstrating its remarkable mechanical strength. In addition, the HEG exhibited excellent adaptability to various deformations, withstanding twisting and folding, and fully recovering its original shape. The material also showed good customizability, enabling fabrication into various geometries tailored for different applications (Fig. 4f).

To further evaluate the elasticity and fatigue resistance of DPF-Zn@LNP-HEG, cyclic tensile tests were performed at different strain levels. As illustrated in Fig. 4g, the stress–strain curves exhibit well-defined hysteresis loops at each strain level, indicating effective energy dissipation through the partial dissociation of non-covalent interactions, including hydrogen bonds, metal coordination bonds, and hydrophobic associations. With increasing strain, both the hysteresis loop area and the energy dissipation capacity increase accordingly. The calculated dissipated energy per cycle increased from 0.04 MJ m^−3^ at 50% strain to 0.30 MJ m^−3^ at 300% strain (Fig. S18), supporting a strain-dependent energy dissipation mechanism governed by the progressive rupture of hydrogen bonds, coordination bonds, and hydrophobic interactions within the HEG network. Furthermore, long-term cyclic tensile tests at a constant strain of 100% were conducted on DPF-Zn@LNP-HEG (Fig. 4h). A decrease in mechanical performance from the second cycle onward reflected the onset of fatigue. This initial stress reduction was attributed to the partial breaking of dynamic hydrogen and coordination bonds during the first cycle, which did not fully recover in subsequent cycles. Notably, the eutectogel maintained excellent mechanical properties even after 100 consecutive cycles, demonstrating outstanding fatigue resistance under repeated deformation. Figure 4j schematically illustrates the energy dissipation mechanism in HEG: during loading–unloading cycles, energy is primarily dissipated through the reversible breaking and reformation of dynamic hydrogen and coordination bonds within the polymer network. Under tension, these bonds dissociate to dissipate energy; upon unloading, they reassociate to restore the network structure. This synergistic energy dissipation mechanism imparts high toughness, rapid recovery, and superior fatigue durability to the HEG. Additionally, as illustrated in Fig. 4i, the HEG samples with a pre-cut notch (5 mm, half of the original width) exhibited excellent notch insensitivity and sustained elongation up to 357%, underscoring their mechanical robustness under severe damage conditions. The fracture energy (Γ) reached 145.08 kJ m^−2^, approximately 14.5 times that of natural rubber (~ 10 kJ m^−2^), demonstrating exceptional resistance to crack propagation [43]. Collectively, these results confirm that DPF-Zn@LNP-HEG achieves enhanced energy dissipation through synergistic sacrificial bonding involving multiple hydrogen bonds and metal coordination interactions. This provides superior mechanical stability and prolonged lifespan for the eutectogel materials.

### Hydrophobic and Anti-Swelling Properties

**Fig. 5:**
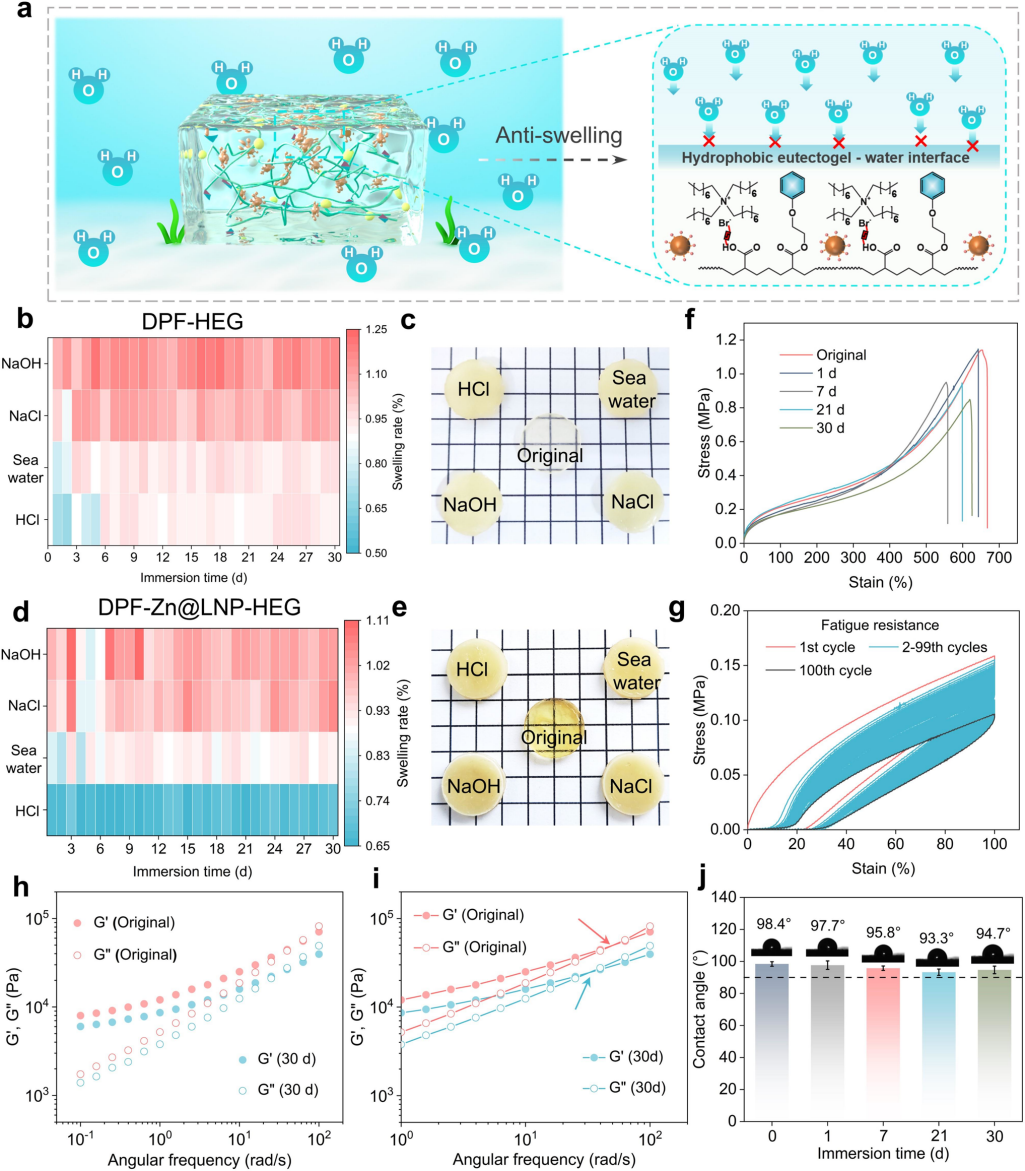
Hydrophobic and anti-swelling properties of DPF-Zn@LNP-HEG. **a** Schematic diagram of the water-repelling mechanism enabled by hydrophobic chains in DPF-Zn@LNP-HEG network. **b–e** Swelling ratios and corresponding photographs of DPF-HEG and DPF-Zn@LNP-HEG after 30 days of immersion in various aqueous media. **f** Stress–strain curves of DPF-Zn@LNP-HEG after seawater immersion for different periods. **g** Cyclic tensile loading–unloading curves of DPF-Zn@LNP-HEG over 100 cycles at 100% strain after 30 days of seawater immersion. **h, i** Frequency-sweep modulus variations of DPF-Zn@LNP-HEG before and after seawater immersion. **j** Water contact angle variations of DPF-Zn@LNP-HEG after seawater immersion for different durations

The anti-swelling capacity of HEG is a critical determinant governing its long-term stability in underwater environments [44]. Building upon our previously developed HEG system, this study systematically investigated its swelling behavior in HCl solution (pH = 1), NaOH solution (pH = 13), NaCl solution (0.9%), and natural seawater environments. Multi-environment immersion tests revealed significant differences in anti-swelling performance between the two HEG materials, DPF-HEG (Fig. 5b) and DPF-Zn@LNP-HEG (Fig. 5d). For DPF-HEG, the equilibrium swelling ratios after 30 days of immersion in different solutions were 0.92% in HCl solution, 0.95% in natural seawater, 1.07% in NaCl solution, and 1.17% in NaOH solution. This excellent anti-swelling performance is attributed to the synergistic interactions between the aromatic rings attached to the phenoxy side chains in the PEA component and the long alkyl chains of TOAB molecules within the material. These interactions form a dense hydrophobic layer with low surface energy through π-π stacking and hydrophobic effects, creating a molecular barrier that effectively resists water penetration and erosion. Interestingly, the incorporation of Zn@LNP reduces the swelling ratio of the DPF-Zn@LNP-HEG by 3.7–22.8% in four different media, with the most significant modification effect observed under acidic conditions. These results indicate that Zn@LNP promotes the self-assembly of nanoscale hydrophobic microdomains within the HEG matrix via hydrogen bonding, metal coordination, and π-π stacking interactions arising from surface aromatic structures. These microdomains interpenetrate with PEA and PHDES to form a compact hydrophobic network, which significantly prolongs the diffusion pathway of water molecules and effectively suppresses swelling (Fig. 5a) [30]. A detailed analysis of performance variations across different environments reveals that the HEG exhibits optimal anti-swelling behavior under acidic conditions. This was attributed to the acidic environment suppressing the dissociation of –COOH groups in the PHDES component, favoring their existence in the molecular state and enhancing hydrogen bonding interactions with TOAB, thereby preserving the structural compactness of the network and further impeding water permeation. In contrast, the swelling ratio in alkaline environments was approximately 47.9% higher than that under acidic conditions. In addition to swelling behavior, notable changes in the macroscopic morphology of HEG were observed. After 30 days of immersion in various environments, both DPF-HEG and DPF-Zn@LNP-HEG changed from their initial transparent state to a uniform milky white appearance (Fig. 5c, e). This phenomenon is analogous to the formation of a milky emulsion when milk is mixed with water. The underlying mechanism originates from the inherent incompatibility between water molecules and hydrophobic polymer segments. When water molecules penetrate the HEG, the hydrophobic segments undergo self-assembly via hydrophobic interactions, leading to the formation of distinct hydrophobic domains. This process subsequently promotes microscopic phase separation, which significantly enhances light scattering [16]. The above research demonstrates that DPF-Zn@LNP-HEG holds considerable potential for underwater applications, owing to its excellent hydrophobicity and anti-swelling performance. To highlight the superior anti-swelling performance of DPF-Zn@LNP-HEG, a control experiment was conducted using a hydrophilic DES-based gel (HDEG) synthesized from AA and ChCl. The HDEG samples were separately immersed in the aforementioned solutions, and the experimental observations revealed the following results: HDEG exhibited distinct swelling characteristics within a short period of time; as the immersion time was extended to 6 h, the HDEG rapidly dissolved and disintegrated subsequently (Figs. S19 and S20). This behavior is in sharp contrast to the stable state of DPF-Zn@LNP-HEG under the same conditions.

However, underwater environments impose more stringent requirements on the mechanical stability of materials. To address this, we further evaluated the mechanical stability of DPF-Zn@LNP-HEG in natural seawater. Experimental data showed that the tensile stress and elongation at break of HEG decrease with prolonged immersion (Fig. 5f). After 30 days of immersion, the tensile strength decreased to 0.85 MPa (74.6% of the initial value), while the elongation at break was maintained at 626% (93.7% of the initial value). This behavior arises from the dynamic sacrificial bond-reconstruction mechanism of hydrogen bonds and coordination bonds within the material. After a small amount of water penetrates the network, water molecules form competitive hydrogen bonds with the polar groups of the polymer chains, replacing the original interchain hydrogen bond cross-linking sites and thereby reducing the effective cross-linking density [5]. In addition, the electrical resistance of DPF-Zn@LNP-HEG decreases during the first week of immersion and then gradually stabilizes. After three weeks of immersion, its conductivity reaches approximately 1.48 × 10^–3^ mS cm^−1^, which is attributed to the intrusion of water and dissociated ions that facilitate charge transport within the material (Fig. S21). Further simulation of natural seawater temperature variations (from 5 to 40 °C) shows that within this range, the ionic conductivity of DPF-Zn@LNP-HEG increases with rising temperature, reaching 1.25 × 10^–3^ mS cm^−1^ at 40 °C, following thermally activated ion migration behavior (Fig. S22). Meanwhile, samples immersed for 21 days at different temperatures maintain good mechanical stability (Fig. S23). These data indicate that although long-term immersion and temperature fluctuations cause slight changes, the core electromechanical performance of HEG remains stable under seawater immersion and variable temperature conditions.

To further investigate the fatigue resistance of DPF-Zn@LNP-HEG under submerged conditions, loading–unloading cyclic tests (100 cycles at 100% strain) were conducted on the sample after 30 days of immersion in natural seawater (Fig. 5g). Compared to the pristine sample, the immersed DPF-Zn@LNP-HEG displayed a significantly smaller hysteresis loop area. This can be attributed to the infiltration of water molecules into the gel network, which reduces the reconstruction efficiency of dynamic hydrogen bonds and prolongs the relaxation time of polymer chains. As a result, more energy is dissipated through irreversible plastic deformation rather than reversible hysteresis processes [45]. Nevertheless, the energy dissipation of DPF-Zn@LNP-HEG remained nearly unchanged throughout the cyclic tests, demonstrating its exceptional underwater fatigue resistance. Rheological tests further revealed that both the storage modulus (G′) and loss modulus (G″) of DPF-Zn@LNP-HEG exhibited a slight decrease with prolonged immersion time (Fig. 5h). The gradual disruption of the cross-linked network, weakened intermolecular interactions, and cumulative swelling effects caused by water molecules collectively prolong polymer chain relaxation time, ultimately shifting the crossover point of G′ and G″ toward lower frequencies (Fig. 5i). These results underscore the influence of seawater environments on the mechanical properties of HEG, offering valuable insights for the structural optimization of DPF-Zn@LNP-HEG in underwater applications. To further assess the water resistance of DPF-Zn@LNP-HEG, the material was immersed in water for 30 days. After immersion, the surface water contact angle slightly decreased from 98.4° to 94.7°, yet the material retained excellent hydrophobicity (Fig. 5j). This phenomenon can be ascribed to the dynamic equilibrium between the hydrophobic surface groups of HEG and water molecules. During immersion, a small amount of water molecules adsorbed on the HEG surface, leading to a slight reduction in the contact angle. However, the internal hydrophobic regions and dynamic cross-linking network effectively preserved the stability of the overall hydrophobic interface. This suggests that the hydrophobic surface of DPF-Zn@LNP-HEG can effectively resist degradation in the natural seawater environment, offering significant advantages for applications such as underwater sensing and marine biological monitoring [46].

### Adhesion Properties

**Fig. 6:**
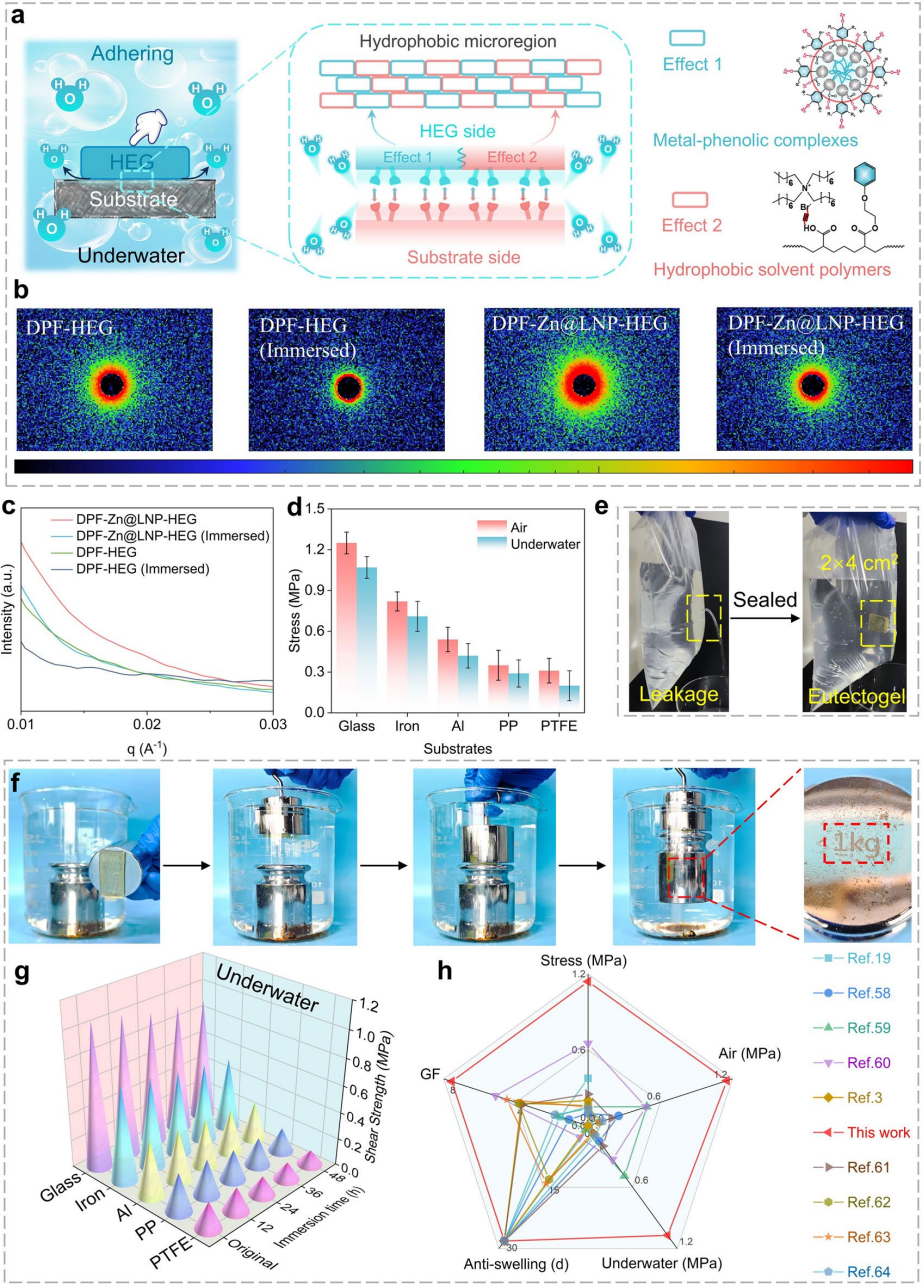
Adhesion performance of DPF-Zn@LNP-HEG. **a** Schematic diagram of self-hydrophobization process and hydrophobic microdomains. **b** 2D SAXS patterns and **c** SAXS spectra of DPF-HEG/DPF-Zn@LNP-HEG before and after immersed in water. **d** Comparison of adhesion strengths on different substrates in air and underwater. **e** Photo images of HEG for sealing material. **f** Instantaneous underwater adhesion on an iron substrate. **g** Evolution of shear strengths after immersion in seawater for different durations. **h** Radar chart comparing the HEG’s anti-swelling ability, tensile strength, air/underwater adhesion, and gauge factor with other reported eutectogels

The HEG system designed in this study not only exhibits excellent mechanical properties and anti-swelling performance but also possesses unique self-adhesiveness, making it highly promising for underwater applications. To explore this potential, we systematically evaluated the interfacial adaptability of HEG by comparing its adhesive performance on various substrates in both air and aqueous environments. From a molecular perspective, the abundance of polar groups within HEG, such as carboxyl, hydroxyl, quaternary ammonium, amide, and carbonyl groups, provides the chemical foundation for its adhesive properties. Experimental results indicated that the adhesion strength of HEG increases with rising DMF content (Fig. S24). However, this change involves a trade-off: excessive DMF compromises the tensile properties and anti-swelling performance of HEG. Therefore, the HEG sample containing 4 wt% DMF was selected for further applications after comprehensive evaluation. In adhesion tests within an air environment, HEG exhibited exceptional adhesive performance. Specifically, after 10 min of static contact on a 2 × 2 cm^2^ glass substrate, the HEG sample was able to sustain a 5.0 kg load without detachment, underscoring its high load-bearing capacity (Fig. S25 and Movie S2). To more precisely quantify the adhesion strength of HEG, lap-shear tensile tests were conducted. The results showed that DPF-Zn@LNP-HEG exhibited excellent adhesive adaptability and considerable bonding strength across multiple substrates, with measured adhesion strengths of 1.25 MPa on glass, 0.82 MPa on iron, 0.54 MPa on aluminum (Al), 0.35 MPa on polypropylene (PP), and 0.31 MPa on polytetrafluoroethylene (PTFE) (Figs. 6d and S26). Notably, DPF-Zn@LNP-HEG retained 57% of its initial lap-shear adhesion strength even after 10 repeated adhesion cycles, highlighting its good reusability (Fig. S27). Microscopic analysis revealed that the robust adhesion between HEG and substrates stems from the synergistic interplay of chemical and physical interactions. These interactions primarily include the directional binding of hydrogen bonds, strong interactions of metal coordination, electrostatic attraction derived from cation-π interactions, as well as the combined effects of ionic and dipole–dipole interactions (Fig. S28) [43, 47].

Driven by the demands for waterproof flexible electronics, this study further investigated the underwater adhesion performance of HEG. As shown in Fig. 6d, HEG exhibits excellent underwater adhesion performance, with an adhesion strength of 1.07 MPa on glass substrate. In practical scenarios of underwater leakage prevention, HEG can rapidly seal damaged packaging, instantly halting water leakage (Fig. 6e and Movie S3). Furthermore, Fig. S29 and Movie S4 show that even when submerged, HEG maintains strong adhesion to the above-mentioned multiple substrates and can withstand externally applied forces from different directions. Notably, on a smooth iron substrate, HEG was able to instantly lift a 1.0 kg weight without requiring any external stimulus, highlighting its robust underwater adhesive capability (Fig. 6f and Movie S5). Underwater adhesion failure is typically caused by the hydration layer formed on substrate surfaces, which prevents intimate contact between the adhesive material and the substrate. However, the benzene ring-containing PEA component within the HEG network disrupts the hydrogen bonding network among water molecules at the interface, thereby breaking down this hydration layer [46, 48]. Simultaneously, the hydrophobic microdomains are synergistically constructed through the metal-phenolic complexation effect and the hydrophobic solvent-polymer effect promotes the expulsion of water molecules from the interface when HEG contacts the substrate. This enables HEG to function effectively in aqueous environments and achieve strong adhesion (Fig. 6a). The 2D SAXS patterns (Fig. 6b) show that DPF-Zn@LNP-HEG exhibited stronger and broader scattering halos, with scattering intensity significantly higher than that of DPF-HEG at lower q values, indicating that the introduction of Zn@LNP enhances low-angle scattering, promotes synergistic assembly with the hydrophobic solvent and polymer network, and leads to the formation of enriched hydrophobic microdomains. After water immersion, the central scattering of DPF-HEG is markedly diminished, while DPF-Zn@LNP-HEG still retains strong scattering signals, demonstrating that its structure remains stable in the aqueous environment. This indicates that Zn@LNP not only acts as a nucleation center for the aggregation of hydrophobic components to facilitate microdomain formation, but also enhances interfacial binding through Zn^2+^ coordination, thereby improving the water stability of the hydrophobic microdomains. The SAXS spectra further reveal that DPF-Zn@LNP-HEG exhibits the highest scattering intensity in the low q region (0.01–0.02 Å^–1^), and this signal remained significantly higher than that of DPF-HEG (Immersed) after immersion (Fig. 6c). Enhancement in the low q region typically corresponds to nanoscale phase-separated domains, so these features can be attributed to the hydrophobic microdomains formed within the system [49]. In summary, Zn@LNP induces and stabilizes the hydrophobic microdomains inside the gel, which serve as the key structural basis for the material’s excellent water resistance, underwater adhesion performance, and long-term stability. In practical tests, the adhesion of HEG remained stable on various substrates in water, even under a 5.0 kg weight load (Fig. S30 and Movie S6). The test results showed that, although a slight decline in adhesion strength was observed for DPF-Zn@LNP-HEG after 48h of immersion in water, it still retained high adhesion strength across various substrates (Fig. 6g).

### Underwater Sensing Properties

**Fig. 7:**
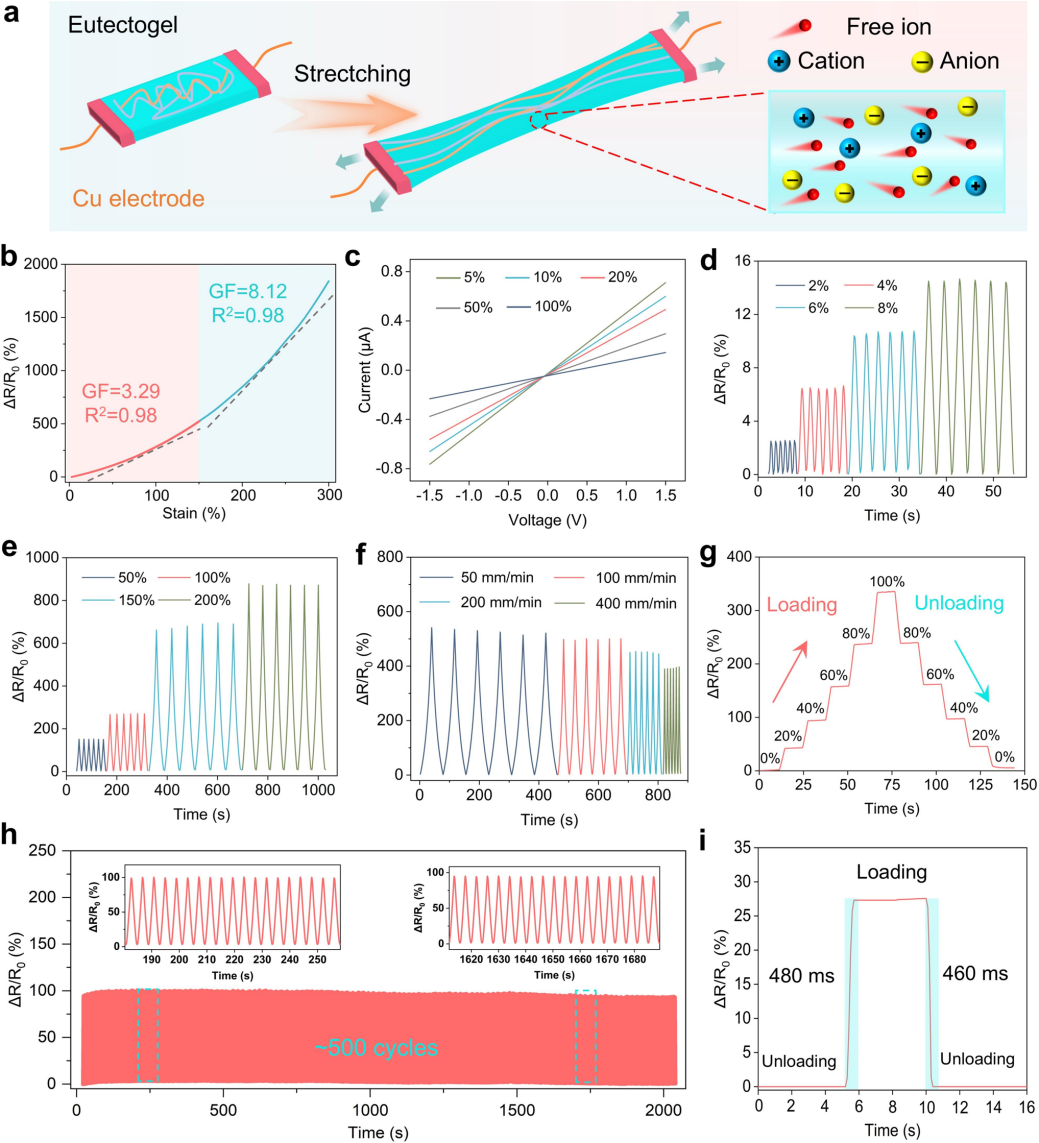
Strain-sensing performance of DPF-Zn@LNP-HEG. **a** Schematic of the eutectogel-based strain sensor. **b** Δ*R*/*R*_*0*_ as a function of applied strain (0–300%) underwater. **c** Current–voltage (I-V) curves under tensile strains from 5 to 100%. **d, e** Δ*R*/*R*_*0*_ under small (2–8%) and large strains (50–200%). **f** Δ*R*/*R*_*0*_ at strain rates of 50–400 mm min^−1^ (100% strain). **g** Δ*R*/*R*_*0*_ under stepwise strain (0–100%). **h** Cycling stability tested at 50% strain and stretching speed of 500 mm min^−1^. **i** Response and recovery times

In recent years, eutectogels have garnered widespread attention due to their ability to effectively integrate with electronic components for monitoring biomechanical signals [50–52]. Owing to its outstanding mechanical properties, exceptional anti-swelling behavior, and robust underwater adhesion, DPF-Zn@LNP-HEG was selected for the development of a flexible wearable device. All HEG samples were immersed in seawater for 30 days to simulate real underwater conditions. The sensitivity of the HEG was evaluated using the gauge factor (GF), calculated from the slope of the relative resistance change (Δ*R*/*R*_*0*_) versus strain [53]. As illustrated in Fig. 7b, Δ*R*/*R*_*0*_ increases with stretching strain. This phenomenon is attributed to the reduction in the eutectogel’s cross-sectional area during stretching, which increases resistance by impeding ion transport (Fig. 7a) [54]. The calculated GF increases with tensile strain, reaching 3.29–8.12 in the strain ranges of 0–150% and 150–300%, respectively, indicating that the sensor exhibits high sensitivity over a wide strain range. As compared with unsoaked samples, the GF values remain essentially stable throughout the immersion period, which showed only slight fluctuations (Fig. S31), which is mainly attributed to ion erosion and molecular chain relaxation within the gel network induced by prolonged seawater exposure [55, 56]. Figure 7c depicts the variations in the current–voltage (I-V) curves of the strain sensor as the tensile strain increases from 5% to 100%. The I-V curves remained linear between − 1.5 and 1.5 V, and the slope of the curve decreased with increasing strain, confirming a strong correlation between resistance variation and applied strain.

To further confirm the sensor’s operational stability, real-time *ΔR/R*_*0*_ measurements were conducted to assess its electromechanical performance. The DPF-Zn@LNP-HEG strain sensor accurately distinguished between different levels of deformation. Figure 7d, e shows the Δ*R*/*R*_*0*_ response under small strain (2–8%) and large strain (50–200%). The results demonstrate that Δ*R*/*R*_*0*_ is closely correlated with strain, exhibiting stable and reversible responses. The sensor’s repeatability was also evaluated at different stretching speeds (50–400 mm min^−1^) (Fig. 7f). The data showed that the DPF-Zn@LNP-HEG produced stable and repeatable resistance signals, demonstrating high reliability. Additionally, a stepwise strain test with incremental deformation (0–100%) demonstrated the HEG’s excellent stability and recovery capability (Fig. 7g). During the loading–unloading process, the Δ*R*/*R*_*0*_ value remained consistent at each strain, which is attributed to the pre-stretching process facilitating faster polymer chain contraction along the directional axis [57]. Response time is a critical performance parameter for flexible wearable devices. When attached to a volunteer’s finger joint, the DPF-Zn@LNP-HEG showed a response time of 480 ms and a recovery time of 460 ms during finger flexion, enabling real-time monitoring of human motion (Fig. 7i). These results collectively demonstrate that the DPF-Zn@LNP-HEG strain sensor, with its wide strain range, high sensitivity, excellent electromechanical stability, rapid dynamic response, and cyclic reliability, demonstrates outstanding underwater sensing performance. To highlight the unique design and superior performance of the DPF-Zn@LNP-HEG strain sensor, Fig. 6g and Table S1 compare it with eight representative eutectogel-based underwater sensors from recent literature. This sensor demonstrates exceptional advantages across five key metrics: tensile strength, adhesion in both air and underwater, anti-swelling capacity, and GF [3, 19, 58–64]. It thus offers a high-performance sensing solution for applications in marine engineering, underwater flexible electronics, and other related fields.

### Underwater Communication and Motion Detection Properties

**Fig. 8:**
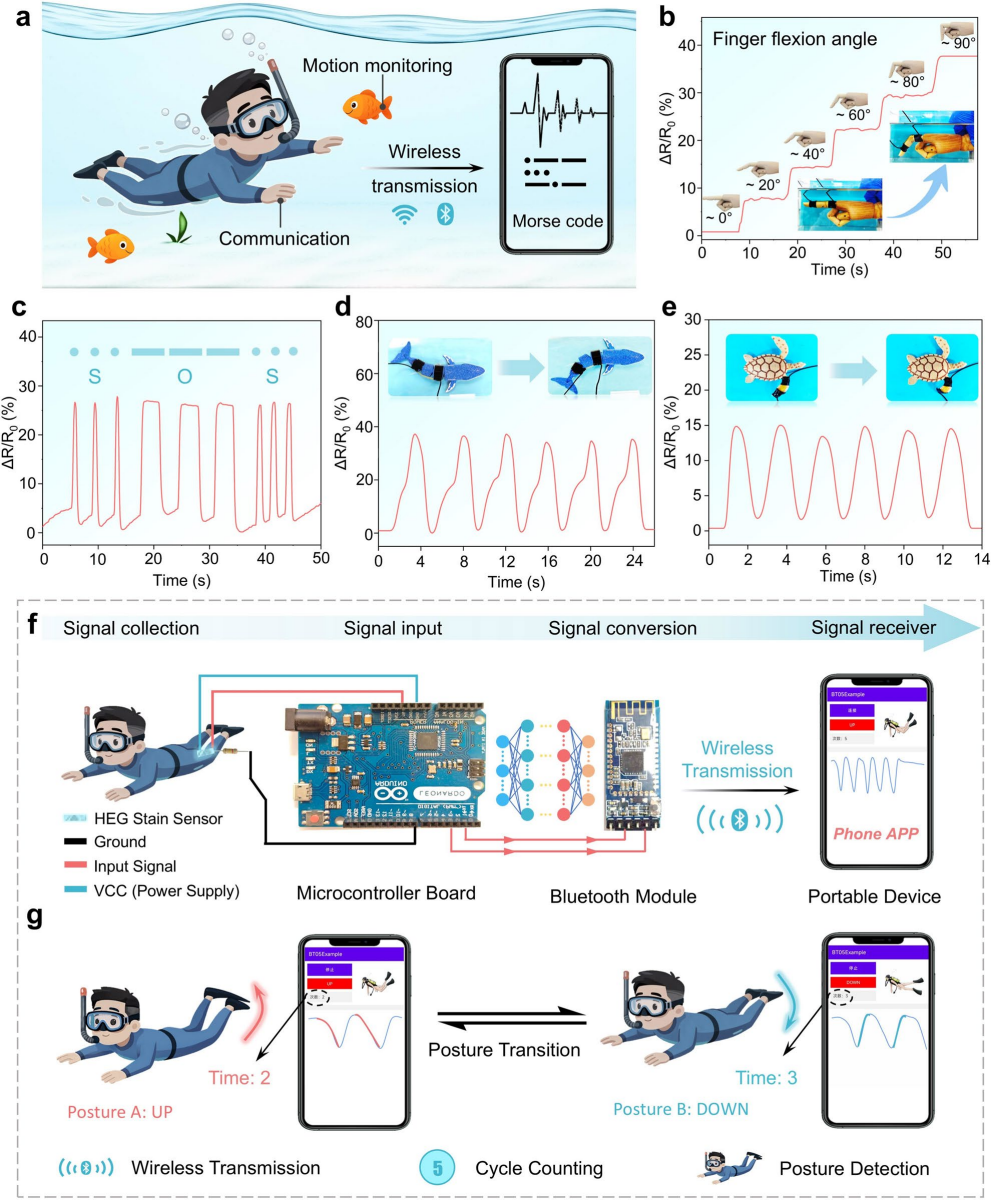
Underwater communication and motion monitoring applications of DPF-Zn@LNP-HEG. **a** Schematic for underwater communication and motion monitoring applications. **b** Real-time Δ*R*/*R*_*0*_ versus finger bending angles. **c** Real-time Δ*R*/*R*_*0*_ for the Morse code signals of “SOS”. Δ*R*/*R*_*0*_ recorded from the sensor attached to the **d** robotic whale and **e** turtle models. **f** Operation and principle of the human–computer interactive swimming posture detection system. **g** Real-time monitoring and counting of swimming motions via the wireless sports assistant system

Owing to its high sensitivity, rapid response characteristic, and long-term sensing stability, the DPF-Zn@LNP-HEG strain sensor exhibits significant potential for various underwater applications. Prior to testing, we evaluated the in vitro* cytotoxicity* of the material using L929 mouse fibroblast cells. As shown in Fig. S32, even at 100% sample concentration, the cell viability remained above 94.5% after 24 h of treatment with the DPF-Zn@LNP-HEG extract. This indicates that the material possesses excellent biocompatibility, which strongly supports its safe and stable attachment to human skin. In a simulated underwater human–computer interaction experiment, the HEG sheet was precisely attached to the surface of the finger joint, coated with a waterproof sealing layer, and then submerged in seawater (Fig. 8a). As the finger gradually bends from its natural extended position (0°) to 90°, the ionic conductive network within the HEG deforms under strain, causing a uniform reduction in the cross-sectional area of the conductive channels [65]. Results in Fig. 8b show that the Δ*R*/*R*_*0*_ value increased steadily by approximately 6% for every 20° increase in bending angle, providing a highly repeatable response that serves as a quantitative benchmark for capturing the movements of humans or animals underwater. Furthermore, the HEG sensor demonstrated the capability for encrypted information transmission via Morse code. This code represents alphabetic characters through specific sequences of dots and lines (Fig. S33). By customizing a signal processing module, the sensor’s rapid deformation (within 2s) was encoded as a “dot” signal in Morse code, represented by a sharp peak in the resistance signal, while sustained deformation lasting 5 s was interpreted as a “dash (—)” signal, corresponding to a plateau in the resistance signal (Fig. S34). In a simulated underwater communication scenario, a volunteer successfully transmitted the “SOS” emergency signal via finger bending under seawater conditions (Fig. 8c). Similarly, in Morse code experiments for the words “FISH” (Fig. S35a) and “WHALE” (Fig. S35b), finger bending movements enabled seamless signal transmission, confirming the practical potential of the HEG sensor for underwater Morse code communication.

Additionally, the DPF-Zn@LNP-HEG can also function as a marine strain sensor for long-term monitoring of marine animal movements. The sensor, which consists of a HEG component, insulating tape, and connecting wires, was affixed to the tail joint of a robotic whale model and the forelimb base of a bionic sea turtle model for subsequent experiments. During the whale’s swimming motion, the motor-driven tail swings sideways to generate forward thrust. The resulting resistance signal exhibited a characteristic signal profile consisting of a main peak and a shoulder peak. The main peak corresponds to the maximum strain when the tail fin is fully flexed, whereas the shoulder peak is triggered by the elastic rebound strain when the movement direction reverses (Fig. 8d). In contrast, the amplitudes of the peaks and valleys generated by the up-and-down movement of the sea turtle’s foot were nearly identical, likely due to the smaller strain produced during its motion (Fig. 8e). From a signal analysis standpoint, the movement states of marine animals can be inferred based on signal intensity and periodic features. As illustrated in Fig. S36, as the swing amplitudes of the sea turtle forelimb increase, the Δ*R*/*R*_*0*_ value shows a significant upward trend. This can be attributed to larger swing amplitudes result in more pronounced stretching deformation of the eutectogel, which in turn leads to a more noticeable change in cross-sectional area and thus greater resistance to ion transport. These results confirm a strong positive correlation between swing amplitude and relative resistance change, highlighting the suitability of the DPF-Zn@LNP-HEG strain sensor for tracking dynamic movements and physiological signals in marine animals, thereby positioning it as a promising tool for long-term monitoring of marine animal behavior. Moreover, the sensor produces consistent signals in both air and underwater environments, demonstrating wide applicability and excellent environmental stability (Fig. S37a, d).

To further validate the practical applicability of the strain sensor in underwater environments, it was integrated into a visualized sensing system. A wireless motion assistance system equipped with a custom mobile application (APP) was also constructed based on this intelligent recognition unit. Detailed structural connections and logic circuit diagrams are provided in Figs. 8f and S38, respectively. When under external strain, the sensor exhibits systematic changes in its intrinsic resistance. These changes are converted into measurable voltage signals, which are first captured in real time by a signal acquisition module. After signal conversion and amplification via an operational amplifier (OPA), the data are transmitted through a Bluetooth module over a stable wireless link to portable devices such as smartphones, where they are displayed as real-time waveforms. The sensor can be attached to an athlete’s leg to monitor swimming posture and count leg kicks. During swimming, the APP analyzes leg flexion signals and displays a visualized voltage change curve on the smartphone screen (Fig. 8g and Movie S7). The red and blue segments of the curve correspond to upward and downward leg movements, respectively. Based on the distribution of these signal segments, users can assess the athlete’s swimming posture in real time. Furthermore, the repetition frequency and intervals of the signal curve can be used to statistically determine kick frequency and count, enabling real-time evaluation of swimming performance. These results not only confirm the reliable operation of the DPF-Zn@LNP-HEG strain sensor in complex underwater environments but also highlight its significant potential for applications such as human motion monitoring and marine organism behavior observation.

## Conclusion

This study presents a multifunctional hydrophobic eutectogel (DPF-Zn@LNP-HEG) fabricated via one-step UV-induced polymerization. Its architectural design is based on a pioneering, bifunctional polymerizable hydrophobic deep eutectic solvent (PHDES) as the matrix, with 2-phenoxyethyl acrylate (PEA) integrated to further enhance the intrinsic hydrophobicity, Zn^2+^-coordinated lignin nanoparticles (Zn@LNP) incorporated as reinforcing fillers to strengthen mechanical properties, and *N,N*-dimethylformamide (DMF) used to optimize interfacial interactions. This design strategy achieves enhanced performance through three synergistic mechanisms. First, the polymerizable double bonds and long alkyl chains in PHDES co-assemble into a three-dimensional network architecture that combines hydrophobicity with reactivity. Second, the hydrophobic microdomains, formed by hydrophobic polymer networks and metal-phenolic complexes (Zn@LNP), effectively disrupt the hydration layer and impede water penetration. Meanwhile, Zn@LNP act as dynamic sacrificial cross-linkers, significantly enhancing mechanical strength, energy dissipation, and anti-swelling properties. Third, DMF serves as an interfacial modulator, enhancing the compatibility of HEG with diverse substrates and notably improving underwater interfacial contact and adhesion strength. The resulting eutectogel exhibits a comprehensive set of excellent properties: high mechanical strength (1.14 MPa), exceptional toughness (3.15 MJ m^−3^), superior anti-swelling properties (< 1% after 30 days), robust underwater adhesion (1.07 MPa), high strain sensitivity (GF = 8.12), and reliable underwater sensing capability. These synergistic advantages position the HEG as a versatile platform for underwater sensing applications, including Morse code transmission, biomotion monitoring, and Bluetooth-enabled wireless swimming motion tracking. Overall, this work proposes a novel strategy for designing hydrophobic gel materials with integrated multifunctional underwater performance, laying a material foundation for the development of next-generation flexible marine electronics.

## Supplementary Information

Below is the link to the electronic supplementary material.

Supplementary file1 (MP4 5211 KB)

Supplementary file2 (MP4 4282 KB)

Supplementary file3 (MP4 4710 KB)

Supplementary file4 (MP4 8600 KB)

Supplementary file5 (MP4 4621 KB)

Supplementary file6 (MP4 3720 KB)

Supplementary file7 (MP4 3807 KB)

Supplementary file8 (DOCX 10522 KB)
