# Supplementary material for: Hydrophobic Eutectogels Reinforced by Zn2+-Coordinated Lignin Nanoparticles for Underwater Wearable Electronics
Source: Nanomicro Lett. 2026 Aug 3;19:20. doi: 10.1007/s40820-026-02302-9 (PMC13433710; doi:10.1007/s40820-026-02302-9)
Supplement: Supplementary file 1 [file 40820_2026_2302_MOESM8_ESM.docx]

Supporting Information for

**Hydrophobic Eutectogels Reinforced by Zn^2+^-Coordinated Lignin Nanoparticles for Underwater Wearable Electronics**

Fuyuan Lu ^1, 2^, Dan Sun ^2, 3,^ *, Wei He ^1^, Meng Li ^1^, Zhengjun Shi ^3, 4^, Yiqiang Wu ^5^, Shao-Fei Sun ^1,^ *, Changyou Shao ^1,^ *, Runcang Sun ^1,^ *

^1^ Liaoning Key Laboratory of Lignocellulose Chemistry and Biomaterials, College of Light Industry and Chemical Engineering, Dalian Polytechnic University, Dalian 116034, P. R. China

^2^ College of Chemistry and Materials Engineering, Zhejiang A&F University, Hangzhou 311300, P. R. China

^3^ The Key Laboratory of Forest Resources Conservation and Utilization in the Southwest Mountains of China Ministry of Education, Key Laboratory of National Forestry and Grassland Administration on Biodiversity Conservation in Southwest China, Yunnan Provincial Key Laboratory for Conservation and Utilization of In-forest Resource, Southwest Forestry University, Kunming 650224, P. R. China

^4^ College of Materials and Chemical Engineering, Southwest Forestry University, Kunming 650224, P. R. China

^5^ College of Materials Science and Engineering, Central South University of Forestry and Technology, Changsha 410004, P. R. China

*Corresponding authors: [sunday4556@zafu.edu.cn](mailto:sunday4556@zafu.edu.cn) (Dan Sun); [sunshaofei153@163.com](mailto:sunshaofei153@163.com) (Shao-Fei Sun); [shaocy@dlpu.edu.cn](mailto:shaocy@dlpu.edu.cn) (Changyou Shao); [rcsun3@dlpu.edu.cn](mailto:rcsun3@dlpu.edu.cn) (Runcang Sun)

**S1 Supplementary Text**

***S1.1 Preparation of DP******-HEG***

The D_2_P_3_-HEG was synthesized as a representative example using the following procedure: 2.0 g PHDES, 3.0 g PEA, and 50 mg I2959 were mixed with stirring for 1 h until a homogeneous solution formed. Subsequently, the mixture was carefully transferred into a polytetrafluoroethylene (PTFE) mold (80 × 20 × 2 mm^3^ internal dimensions) and photopolymerized under UV light for 30 min to obtain copolymer eutectogels.

***S1.2 Preparation of DPF-HEG***

The D_2_P_3_F_4_-HEG was synthesized as a representative example using the following procedure: 2.0 g PHDES, 3.0 g PEA, 0.2 g DMF (4 wt% for the total monomers), and 52 mg I2959 were mixed and photopolymerized into a eutectogel under UV light.

***S1.3 Characterization Methods***

Chemical structures of the samples were analyzed by Fourier-transform infrared (FT-IR) spectrometer (PerkinElmer, Japan). Each dried sample was mixed with KBr and scanned 64 times over a wavenumber range of 4000-400 cm^-1^. Further chemical analysis was performed by X-ray photoelectron spectroscopy (XPS) (Thermo Scientific K-Alpha, U.S.A.). Proton nuclear magnetic resonance (^1^H NMR) spectra were recorded on a Bruker AVIII (400 MHz) spectrometer with samples (30 mg) dissolved in DMSO‑*d6* (0.6 mL). The optical transparency of HEG was evaluated from 800 to 400 nm using a UV-vis-NIR spectrophotometer (Hitachi, Japan). Differential Scanning Calorimetry (DSC) measurements of TOAB, AA, and PHDES were conducted on a TA DSC 250thermal analysis system (Waters, Shanghai) under N_2_ atmosphere at a rate of 10 °C min^-1^. The particle size distribution of Zn@LNP complexes was determined using a Zetasizer Nano ZS (Malvern, U.K.). Water contact angles were measured on a JC2000D surface contact angle goniometer (Powereach, China) by depositing a 30 μL of water droplet onto the eutectogel surface. Cross-sectional morphology of the eutectogel was examined by field emission scanning electron microscopy (FE-SEM, JSM-7800F, Japan) operated at an accelerating voltage of 4 kV, coupled with energy-dispersive X-ray spectroscopy (EDS). Prior to imaging, the lyophilized samples were quenched in liquid nitrogen and sputter-coated with gold. Elemental mapping was performed based on EDS elemental analysis. Rheological characterization of the samples was performed using a TA Instruments AR-G2 rheometer equipped with a 40 mm diameter, 1° cone-plate geometry. A frequency-sweep test was conducted in which the frequency was raised from 0.1 to 100 Hz and the strain was held constant at 1%. 1D small-angle X-ray scattering (SAXS) profiles and 2D SAXS patterns were acquired on a Xeuss 2.0 system (France) equipped with a Pilatus 3R 300K semiconductor detector, with an X-ray wavelength of 1.54189 Å.

***S1.4 Mechanical Test***

Mechanical properties of different eutectogels were evaluated using a universal testing machine (Instron 5965) with a 500 N load cell at room temperature. Rectangular specimens (40 × 10 × 2 mm^3^) underwent uniaxial tensile tests at a constant crosshead speed of 100 mm·min^-1^ with ends clamped securely. All measurements were performed in triplicate to ensure reproducibility. The fracture stress and strain were directly determined from the stress-strain curve at fracture. The toughness was calculated as the integral area under the stress-strain curve. Fatigue resistance was assessed through cyclic tensile tests under identical loading conditions.

***S1.5 Swelling Test***

The swelling properties of the eutectogels were evaluated by monitoring the swelling ratio changes over a period of 30 days in NaOH (pH=13), NaCl (0.9%), HCl (pH=1), and natural seawater. The weight of each sample was recorded daily. To simulate the real water flow, the NaOH, NaCl, HCl, and natural seawater used for swelling were replaced daily. The swelling ratio was calculated using the following equation:

$$Swelling ratio \left( \% \right)=\frac{w_{s}}{w_{0}}-1$$

where w_s_ and w_0_ were the weight of swollen hydrogel and original eutectogel, respectively.

***S1.6 Adhesion Test***

Adhesion strength was measured via lap-shear testing. Briefly, the eutectogel samples (20 × 20 × 2 mm^3^) were sandwiched between two substrates under a preload for 10 min to ensure complete interfacial contact between the HEG and the substrates. Subsequently, the substrates were pulled apart at a rate of 100 mm·min^-1^ using a universal testing machine to determine the adhesive strength.

In the underwater adhesion test, the eutectogel samples were directly bonded between two substrate pieces underwater. For the long-term underwater repeated adhesion test, the specimen was first immersed in the aqueous environment for a specified number of times, then placed between two substrate pieces underwater and subjected to a preload held for 10 min, followed by removal from water for testing.

***S1.7 Sensing Test***

The DPF-Zn@LNP-HEG was assembled into a strain sensor for operation in natural seawater to assess its sensing properties underwater, copper foils were wrapped with waterproof tape, and the testing region was submerged for measurements. Real-time resistance (R_t_) of the sensor was derived from the current-time (i-t) curve recorded by an electrochemical workstation (CS2350M, CorrTest Instruments Corp., Ltd.) under a constant applied voltage of 5 V. The relative resistance change (ΔR/R_0_) was calculated using the following equation:

$$\Delta R/R_{0}=\frac{R_{t}-R_{0}}{R_{0}}$$

where R_0_ and R_t_ denote the initial resistance in the unstretched state and real-time resistance in the stretched state of the sensor, respectively.

The gauge factor (GF), reflecting the strain sensitivity of the sensor, was determined as:

$$GF=\frac{{\Delta R}/{R_{0}}}{\varepsilon}$$

where ε is the applied tensile strain.

***S1.8 In vitro cytotoxicity***

L929 cells were cultured in DMEM high glucose medium (Viva cell, QS-S423) supplemented with 10% fetal bovine serum. Cell viability was assessed using the Cell Counting Kit-8 (CCK-8) assay. Cells were seeded into 96-well plates at a density of 4 × 10^3^ cells per well and incubated overnight at 37 °C in a 5% CO_2_ incubator. Subsequently, the original medium was discarded, and the cells were treated with the sample extract (blank culture medium was used as a control), followed by further incubation for 24 h. Thereafter, 10 μL of CCK-8 reagent was added to each well, and the plates were incubated under light-protected conditions for 3 h. The absorbance at 450 nm was measured using a microplate reader, and the cell viability was calculated.

***S1.9 Density functional theory (DFT) simulations***

All calculations were performed using the Gaussian 16 A.03 software package. The B3LYP functional was adopted for all calculations [S1], in combination with the Grimme’s D3 (BJ) dispersion correction [S2]. For geometry optimization and frequency calculations, the 6-31G* basis set was used for all atoms. The binding energy (*ΔE*) was computed for the optimized molecular system at the B3LYP-D3 (BJ)/def2-TZV level of theory. The *ΔE* is defined as the difference between the energy of the complex and the sum of the energies of the isolated monomers, according to the following equation:

*ΔE=E (system 4)-2E (Zn)-E (A2)-E (B)-E (C)-E (D)-E (E)*

Herein, *ΔE* represents the binding energy, *E* (system 4) signifies the total energy of the whole system 4, and *E* (Zn), *E* (A2), *E (B)*, *E (C)*, *E (D)*, and *E (E)* denotes the total energies of the individual components. Generally, a more negative binding energy is indicative of more robust interactions between the various components.

***S1.10 Statistical Analysis***

All experiments were performed in three times or more (n≥3) for each sample, and the results were reported as the mean ± standard deviation (SD).

***S1.11 Informed consent statement***

All participants voluntarily agreed to participate in the wearable device experiments and provided written informed consent prior to the initiation of any experimental procedures.

**S2 Supporting Figures**

**Fig. S1** Strategy of preparing the Polymerizable Hydrophobic DES (PHDES)

**Fig. S2** The hydrophobicity of TOAB

**Fig. S3** **a** ^1^H NMR spectra and **b** DSC traces of TOAB, AA, and PHDES

**Fig. S4** High-resolution XPS spectra: **a** C1s and **b** O1s regions for AL+ZnCl_2_

**Fig. S5** FT-IR spectra of AL, Ca@LNP, Mg@LNP, Al@LNP, and Fe@LNP

**Fig. S6** Particle size distributions of raw AL

**Fig. S7** EDS elemental mappings of DPF-HEG and DPF-Zn@LNP-HEG

**Fig. S8** FT-IR spectra of PHDES, DP-HEG, DPF-HEG, and DPF-Zn@LNP-HEG

**Fig. S9** Water contact angles of DPF-Zn@LNP-HEG with varied PHDES/PEA mass ratios

**Fig. S10** Transmittance of DPF-HEG and DPF-Zn@LNP-HEG with a thickness of 1 mm in the visible wavelength range (Inset: photograph of DPF-Zn@LNP-HEG)

**Fig. S11** Stress-strain curves of HEG with/without DMF

**Fig. S12** Stress-strain curves of HEG with raw AL, LNP, and Zn@LNP

**Fig. S13** Stress-strain curves of HEG without/with different components

**Fig. S14** **a, b** Particle size distributions of Zn@LNP at various lignin concentrations

**Fig. S15** **a** Stress-strain curves, **b** Young’s modulus, and toughness of Zn@LNP with different particle sizes

**Fig. S16** Particle size distributions of Zn@LNP in the liquid precursor

**Fig. S17** Stress-strain curves of HEG prepared with Zn@LNP, Ca@LNP, Mg@LNP, Al@LNP, and Fe@LNP, respectively

**Fig. S18** Successive cyclic tensile behavior and hysteresis area of DPF-Zn@LNP-HEG at different strains (50-300%)

**Fig. S19** Morphological changes of HDEG after 6 h of immersion in various aqueous media

**Fig. S20** Swelling ratios of HDEG after 6 h of immersion in various aqueous media

**Fig. S21** **a** Electrical impedance and **b** the corresponding ionic conductivity of DPF-Zn@LNP-HEG at different immersion durations

**Fig. S22** **a** Electrical impedance and **b** the corresponding ionic conductivity of DPF-Zn@LNP-HEG at different temperatures

**Fig. S23** Stress-strain curves of DPF-Zn@LNP-HEG after seawater immersion at different temperatures

**Fig. S24** **a** Shear curves and **b** the corresponding adhesion strengths of DPF-Zn@LNP-HEG with different DMF contents on glass substrate

**Fig. S25** Photographs of DPF-Zn@LNP-HEG lifting a 5.0 kg weight

**Fig. S26** Shear curves of DPF-Zn@LNP-HEG on different substrates in **a** air and **b** underwater

**Fig. S27** **a** Shear curves and **b** the corresponding adhesion strengths of DPF-Zn@LNP-HEG on glass substrate at different adhesion cycles. Inset: adhesive interface images of two glass substrates after the first and final adhesion cycles

**Fig. S28** Schematic illustration of the lap-shear test geometry and adhesion mechanisms between DPF-Zn@LNP-HEG and the substrates

**Fig. S29** Photographs illustrating the underwater adhesive behavior of DPF-Zn@LNP-HEG with various substrates

**Fig. S30** Sustained underwater load-bearing capacity on various substrates

**Fig. S31** ΔR/R_0_ as a function of applied strain (0-300%) underwater at different immersion durations

**Fig. S32** Cell viability of L929 cells after 24 h of culture in medium containing different concentrations of DPF-Zn@LNP-HEG extract

**Fig. S33** Schematic diagram of the signal codes

**Fig. S34** Real-time ΔR/R_0_ for the dot (·) and “dash (—)” signals

**Fig. S35** ΔR/R_0_ for the Morse code signals of **a** “FISH” and **b** “WHALE”

**Fig. S36** ΔR/R_0_ versus the swing amplitudes of the robotic turtle’s forelimb

**Fig. S37** **a** Real-time ΔR/R_0_ for the Morse code signals of “SOS”. ΔR/R_0_ recorded from the sensor attached to the **b** robotic whale and **c** turtle models. ΔR/R_0_ versus the swing amplitudes of the robotic turtle’s forelimb

**Fig. S38** The detailed logic circuit of the wireless sport assistant system. The electrical resistance signals from the movement are converted into trigger voltage signals and further modulated by the MCU. The processed signals are then wirelessly transmitted to the APP on personal smartphone by a Bluetooth transmitter, and the sport assessment is finally conducted according to a set procedure

**Table S1** EDS elemental contents of DPF-HEG and DPF-Zn@LNP-HEG

| Element | wt% | Atomic% | Element | wt% | Atomic% |
| --- | --- | --- | --- | --- | --- |
| DPF-HEG | | | DPF-Zn@LNP-HEG | | |
| C | 79.69 | 83.94 | C | 63.24 | 74.70 |
| O | 20.31 | 16.06 | O | 24.20 | 21.46 |
| Cl | 0.00 | 0.00 | Cl | 6.11 | 2.45 |
| Zn | 0.00 | 0.00 | Zn | 6.45 | 1.40 |
| Total | 100.00 | 100.00 | Total | 100.00 | 100.00 |

**Table S2** Comparison of comprehensive properties of representative eutectogel-based underwater sensors from recent literature

| **Sample** | **Stress**  **(MPa)** | **Adhesion**  **(MPa)**  **air** | **Adhesion**  **(MPa) underwater** | **Anti-swelling (Day)** | **Gauge Factor (GF)** | **Ref.** |
| --- | --- | --- | --- | --- | --- | --- |
| PAGDP-Ag | 0.2 | 0.098 | 0.009 | 30 (~1.2%) | 3.98 | [3] |
| HEG | 0.65 | 0.52 | 0.33 | 3 (~1%) | 5.5 | [19] |
| SEG-PIL/M | 0.15 | 0.53 | 0.49 | 1 (~0.06%) | 1.85 | [58] |
| e-PT/GS | 0.014 | NR | 0.354 | 14 (~15%) | 0.44 | [59] |
| Eutectogel | 0.43 | 1.49 | 1.25 | 30 (~80%) | 2.59 | [60] |
| PSA-DA | 0.25 | 0.21 | 0.199 | 30 (~3%) | 3.9 | [61] |
| PHE | 0.16 | 0.101 | 0.082 | 14 (~3%) | 4.14 | [62] |
| SNA-HPC | 0.134 | 0.131 | 0.097 | 30 (~5%) | 1.997 | [63] |
| PAA/SBMA/BC | 0.145 | 0.0206 | 0.0206 | 15 (~15%) | 2.14 | [64] |
| DPF-Zn@LNP | 1.14 | 1.25 | 1.07 | 30 (~1%) | 8.12 | This work |

NR: Not Report

**Supplementary References**

[S1] G. Monaco, F. Aquino, R. Zanasi, W. Herrebout, P. Bultinck et al., Model-averaging of ab initio spectra for the absolute configuration assignment via vibrational circular dichroism. Phys. Chem. Chem. Phys. **19**(41), 28028-28036 (2017). https://doi.org/10.1039/c7cp05358d

[S2] S. Grimme. Density functional theory with london dispersion corrections. WIREs Comput. Mol. Sci. **1**(2), 211-228 (2011). https://doi.org/https://doi.org/10.1002/wcms.30
